# ADAR1 is a new target of METTL3 and plays a pro-oncogenic role in glioblastoma by an editing-independent mechanism

**DOI:** 10.1186/s13059-021-02271-9

**Published:** 2021-01-28

**Authors:** Valentina Tassinari, Valeriana Cesarini, Sara Tomaselli, Zaira Ianniello, Domenico Alessandro Silvestris, Lavinia Ceci Ginistrelli, Maurizio Martini, Biagio De Angelis, Gabriele De Luca, Lucia Ricci Vitiani, Alessandro Fatica, Franco Locatelli, Angela Gallo

**Affiliations:** 1 Oncohaematology Department, IRCCS Ospedale Pediatrico Bambino Gesu, Viale di San Paolo 15, 00146 Rome, Italy; 2 Present address: Department of Molecular Medicine, “Sapienza” University of Rome, Rome, Italy; 3 Present address: Department of Biomedical Sciences, Institute of Translational Pharmacology, National Research Council of Italy (CNR), Rome, Italy; 4 Department of Biology and Biotechnology “Charles Darwin”, La Sapienza University of Rome, Rome, Italy; 5 Department of Women’s, Children’s and Public Health Studies, Fondazione Policlinico Universitario “A. Gemelli”, IRCCS, Largo A. Gemelli 8, 00168 Rome, Italy; 6 Department of Health Science and Public Health, Institute of Pathology, Largo F. vito 1, 00168 Rome, Italy; 7 Department of Oncology and Molecular Medicine, Istituto Superiore di Sanità, Viale Regina Elena 299, 00161 Rome, Italy; 8 Department of Pediatrics, La Sapienza University of Rome, Rome, Italy

## Abstract

**Background:**

*N*^*6*^-methyladenosine (m6A) and adenosine-to-inosine (A-to-I) RNA editing are two of the most abundant RNA modification events affecting adenosines in mammals. Both these RNA modifications determine mRNA fate and play a pivotal role in tumor development and progression.

**Results:**

Here, we show that METTL3, upregulated in glioblastoma, methylates *ADAR1* mRNA and increases its protein level leading to a pro-tumorigenic mechanism connecting METTL3, YTHDF1, and ADAR1. We show that ADAR1 plays a cancer-promoting role independently of its deaminase activity by binding *CDK2* mRNA, underlining the importance of ADARs as essential RNA-binding proteins for cell homeostasis as well as cancer progression. Additionally, we show that ADAR1 knockdown is sufficient to strongly inhibit glioblastoma growth in vivo.

**Conclusions:**

Hence, our findings underscore METTL3/ADAR1 axis as a novel crucial pathway in cancer progression that connects m6A and A-to-I editing post-transcriptional events.

**Supplementary Information:**

The online version contains supplementary material available at 10.1186/s13059-021-02271-9.

## Background

*N*^6^-methyladenosine (m6A) is an important RNA modification mainly occurring at the consensus motif RRm^6^ACH [1, 2]. The identification of m6A modification machinery and the development of transcriptome-wide approaches for m6A sequencing indicated that m6A can affect thousands of coding and non-coding RNAs in a given type of cell. The m6A modification is catalyzed by the m6A methyltransferase complex (MTC), which involves the methyltransferase-like 3 and 14 (METTL3 and METTL14) (i.e., writers) and cofactors (such as the Wilms tumor 1-associated protein or WTAP). The removal of m6A is facilitated by FTO and ALKBH5, two m6A demethylases (i.e., erasers). Additionally, YT521-B (YTH)-like domain family of proteins (YTHDF1, YTHDF2, YTHDF3, YTHDC2, and YTHDC1) have been identified as m6A readers that affect the translation, the stability and/or splicing of target RNAs. Indeed, m6A modification plays essential roles in mRNA stability, splicing, transport, localization, translation, is involved in the primary microRNA processing and RNA-protein interactions, thus being essential in tissue development, self-renewal, stem cell differentiation, DNA damage response and cancer progression [3, 4].

Adenosine-to-inosine (A-to-I) RNA editing is another important co/post-transcriptional event strongly affecting the code of several mRNAs and the structure of dsRNA targets. The A-to-I RNA editing is catalyzed by the adenosine deaminases acting on RNA-1 and -2 (ADAR1/2) in mammals. ADAR-mediated editing, originally identified as distinct A-to-I/G signature mismatch after reverse transcription, now accounts for more than 4 million edited positions [5, 6]. Despite the enormous amount of deaminated adenosines (inosinome) in mammals, the importance of ADAR proteins is also extending as RNA binding proteins [7, 8].

Like METTL3 enzyme, also the ADAR proteins have been indicated as an important player in cancer [9–11]. Herein, we show a direct connection between these two post-transcriptional mechanisms. We report that the ADAR1 protein level is increased by METTL3 and YTHDF1 proteins, both abundantly expressed in glioblastoma (GBM). We found that ADAR1, through its RNA binding domains (RBDs), binds/stabilizes CDK2 a key player in cancer cell-cycle progression, so promoting glioblastoma proliferation in vitro and most importantly in vivo.

Our data reveal the existence of a new pro-tumoral pathway in glioblastoma (METTL3/ADAR1) and indicate ADAR1 as one of the main targets of METTL3 controlling cell proliferation and tumor growth.

## Results

### METTL3 and YTHDF1 increase ADAR1 protein levels

**Fig. 1:**
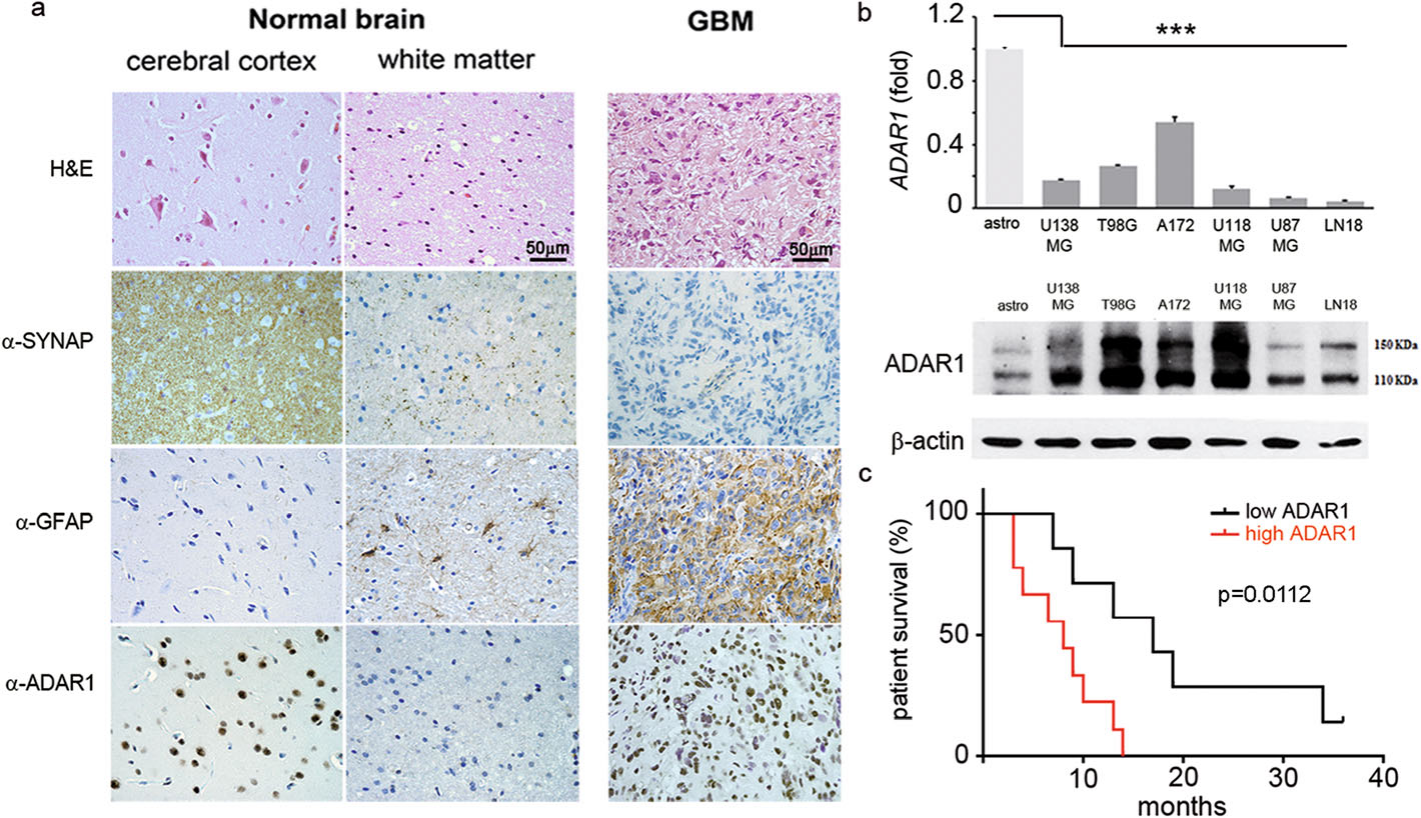
High level of ADAR1 protein is a negative prognostic factor in patients with GBM. **a** H&E and immunohistochemical analysis on GBMs (*n* = 16), cerebral cortex and white matter tissues, showing synaptophysin (neuronal cell marker), GFAP (astrocyte cell marker), and ADAR1 proteins*.* One representative picture of both GBM and control tissues is shown (× 40 magnification), demonstrating that an upregulation of ADAR1 protein is observed in GBM tissues versus an unaltered *ADAR1* mRNA level (Additional file 2, Fig. S1a)*.* **b** Representative qRT-PCR (top panel) and western blotting (bottom panel) analysis of ADAR1 in different glioblastoma cell lines (U138MG, T98G, A172, U118MG, U87MG, LN18) compared to normal astrocytes (*n* = 3). Values are represented as means ± SD, ***p ≤ 0.001. **c** Kaplan-Meier survival plot of GBM patients (*n* = 16) stratified by low (black line) and high (red line) ADAR1 expression. Log-rank test, *p* = 0.0112; HR 3.088, 95% CI 1.050–9.082 (see also Additional file 2, Table 1).

Previous studies have indicated ADAR1 as a key protein in multiple mechanisms controlling normal and pathological cells [11]. Despite so, little is known regarding regulatory pathways controlling the ADAR1 protein levels in normal and cancer tissues. We previously show that *ADAR1* mRNA is not significantly altered in glioblastoma and normal brain [10]. Herein, we report that while ADAR1 mRNA is indeed unchanged, its protein is particularly abundant in cancer tissues (GBM) compared to controls (Fig. 1a, Additional file 1, Fig. S1). Similarly, we observed a strong difference between ADAR1 mRNA and protein levels also in glioblastoma cell lines (U138MG, T98G, A172, U118MG, U87MG, and LN18) compared to primary astrocytes, with a low mRNA amount corresponding to a high ADAR1 protein (Fig. 1b).

Considering the above findings, we first asked whether ADAR1 protein levels could be a prognostic factor for patients’ survival (Kaplan-Meier, 95% confidence). We found that a high protein level of ADAR1 is predictive of a poor patient survival (OS) (log-rank test, *p* value = 0.0112) (Fig. 1c and Additional file 2, Table 1).

Then, in order to analyze the possible reasons of the observed discrepancy between mRNA and protein levels of ADAR1 and considering that a high protein level of ADAR1 is connected with patients’ OS, we investigated whether RNA methyltransferase-like 3 (METTL3) might play a role in controlling ADAR1 protein expression.

Therefore, as the core complex of m6A is formed by METTL3 and METTL14, with the first carrying the catalytic subunit and the latter being an essential factor facilitating the RNA binding, we analyzed the presence/level and cell localization of both METTL3 and METTL14 in GBM.

**Fig. 2:**
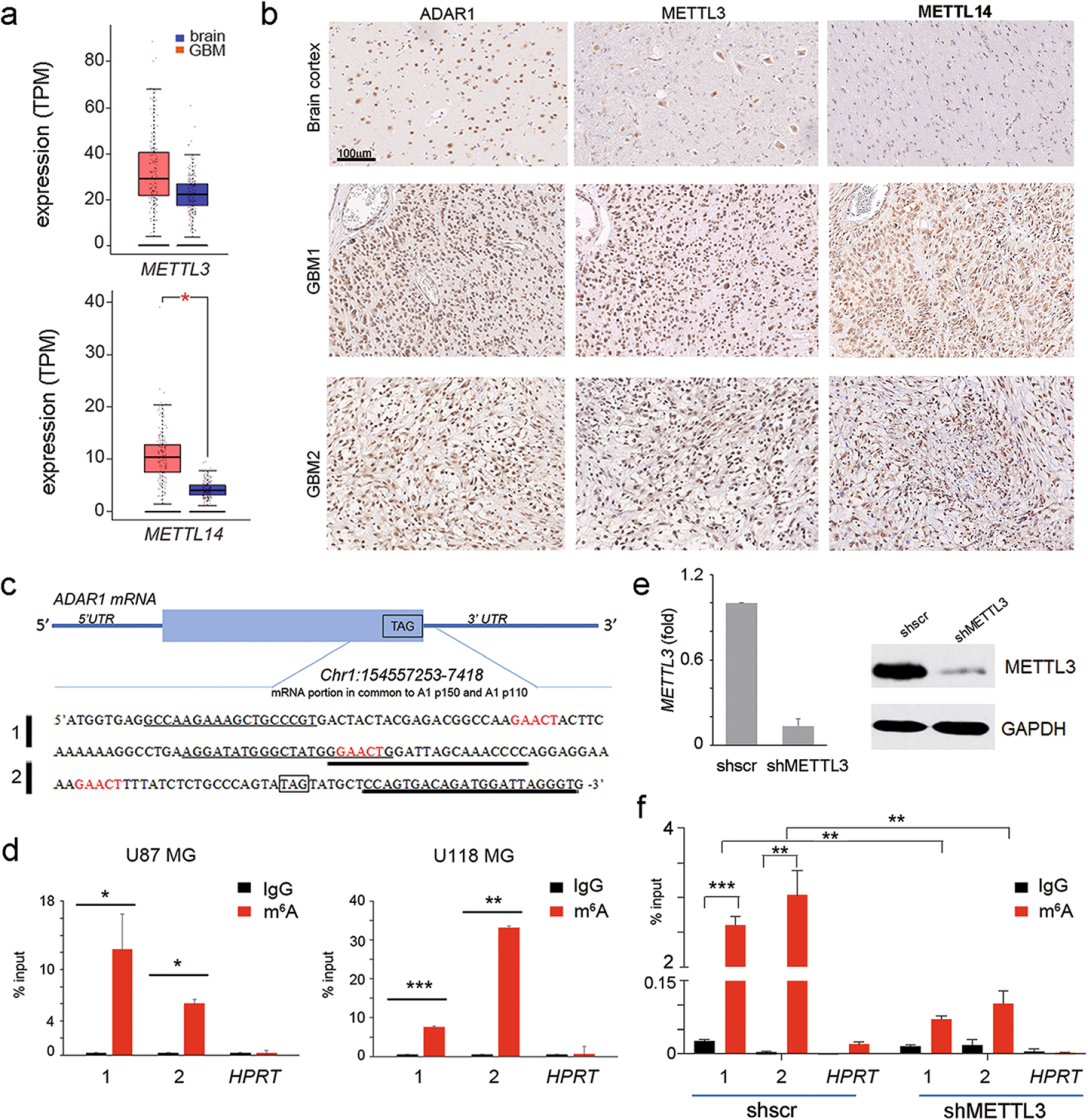
*ADAR1* transcript undergoes m6A modification near the STOP codon in GBM. **a** Box plot showing *METTL3* and *METTL14* mRNA expression in 163 GBMs (red plot) and 207 normal brain cortex tissues (blue plot) (http://gepia2.cancer-pku.cn), * *p* ≤ 0.05. **b** Immunohistochemical analysis performed on 10 GBM tissues and human brain cortex (two representative pictures of GBMs and control are shown). **c** Schematic representation of *ADAR1* mRNA, with details of the RNA sequence near the stop codon (TAG). In red, the m6A consensus sequence within *ADAR1* mRNA. Two different sets of primers for *ADAR1* amplification are shown, with a thin (PCR 1) and a thick (PCR 2) black line. **d** qRT-PCRs (PCR 1 and 2) of *ADAR1* RNA fragments in U87MG (left) and U118MG (right) cells after m6A immunoprecipitation (*n* = 3). *HPRT* expression was used as negative control. **e–f** m6A immunoprecipitation of *ADAR1* mRNA (PCR 1 and 2) and control *HPRT* upon METTL3 silencing (*n* = 3). Values are represented as means ± SD, *p ≤ 0.05; **p ≤ 0.01; ***p ≤ 0.001

We found that both METTL3 and METTL14 are expressed/increased in GBM cells/tissues compared to astrocytes/normal brain cortex with the expected cell localization (Fig. 2a, b, Additional file 1, Fig. S1,2)*.*

Then, by analyzing m6A-seq data performed in several cell lines including glioma stem cells, we observed that, among many, the *ADAR1* transcript showed an enrichment of m6A sites near the stop codon, which is the sequence context frequently methylated in several transcripts [12]. We identified the consensus sequence motif for the m6A methylation, RRACH (R = G or A; H = A, C or U) within *ADAR1* transcripts at multiple sites (GAACU motifs) before the 3′UTR (Fig. 2c), that overlapped with the predicted m6A positions identified in glioma stem cell m6A RIP-seq [13]. Then, to confirm the presence of m6A methylation within *ADAR1* transcript, we used an m6A antibody to immunoprecipitate fragmented RNA isolated from GBM cells (U87MG and U118MG). The qRT-PCR, performed with oligonucleotides specific for *ADAR1* transcript surrounding the m6A methylation motif we identified (PCR 1–2) (Fig. 2c-d), demonstrated the presence of m6A methylation sites in this transcript nearby the stop codon. Then, we modulated METTL3 expression (shMETTL3) in glioblastoma cells and we quantified the m6A-marked sites of ADAR1 by qRT-PCR. Our data demonstrated that the m6A-RNA fragments of ADAR1 (PCR 1–2) were significantly decreased upon METTL3 silencing (Fig. 2e-f). Finally, we downloaded the m6A-seq from shMETTL3/METTL14 and control glioma stem cells [13], and we analyzed the data concentrating on ADAR1 transcript, founding a reduction of ADAR1 methylation levels (including the ones studied in the present study, *Chr1:154557253–7418*) in both the shMETTL3 and shMETTL14 samples (data not shown).

**Fig. 3:**
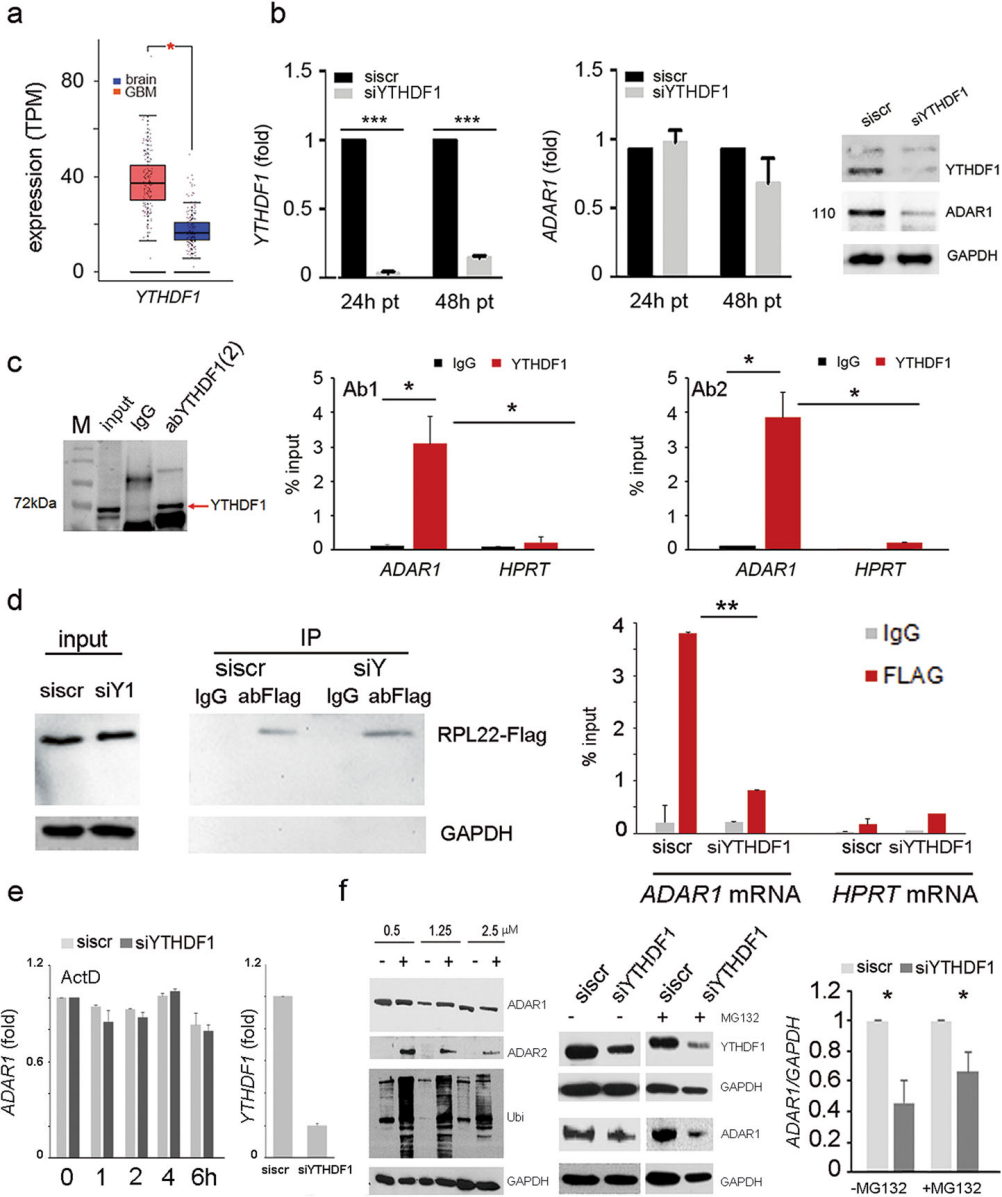
YTHDF1 binds m6A on *ADAR1* mRNA boosting its translation without affecting decay. **a** Box plot showing *YTHDF1* mRNA levels in GBM (red box) versus normal brain (blue box) (GEPIA 2, * *p* ≤ 0.05). **b** qRT-PCR of *YTHDF1* and *ADAR1* in si*YTHDF1* U87MG cells (24–48 h post transfection-pt). On the right, western blotting analysis (48 h pt) of *ADAR1* in si*YTHDF1* U87MG cells is shown. GAPDH was used as control. **c** Relative enrichment of *ADAR1* mRNA in YTHDF1-RIP (using two different antibodies Ab1 and Ab2) over IgG in U87MG cells. *HPRT* expression was used as negative control (*n* = 3) Values are represented as means ± SD, * *p* ≤ 0.05. **d** Ribosomal immunoprecipitation was performed in siYTHDF1 and siscr U87MG cells transfected with an RPL22-FLAG construct. Left, is shown a control western blotting analysis, while on the right, a qRT-PCR is shown. *HPRT* was used as control (*n* = 3). Values are represented as means ± SD, ** *p* ≤ 0.01. **e** *ADAR1* mRNA stability in control and siYTHDF1 U87MG cells was determined by qRT-PCR after actinomycin D (5 μg/ml) treatment in the time indicated. Values are represented as means ± SD. On the right, the qRT-PCR of *YTHDF1* silencing. Values are represented as means ± SD, *** *p* ≤ 0.001, *n* = 2. **f** Western blotting analysis of siYTHDF1 and control U87MG cells treated with or without MG132 at different concentrations (indicated in the figure); ADAR2 and ubiquitin Ab (Ubi) were used as controls. On the right, siYTHDF1 and sictrl U87MG cells were treated with or without 1.25 μM MG132 for 24 h. GAPDH was used as loading control. Values are represented as means ± SD, * *p* ≤ 0.05

**Fig. 4:**
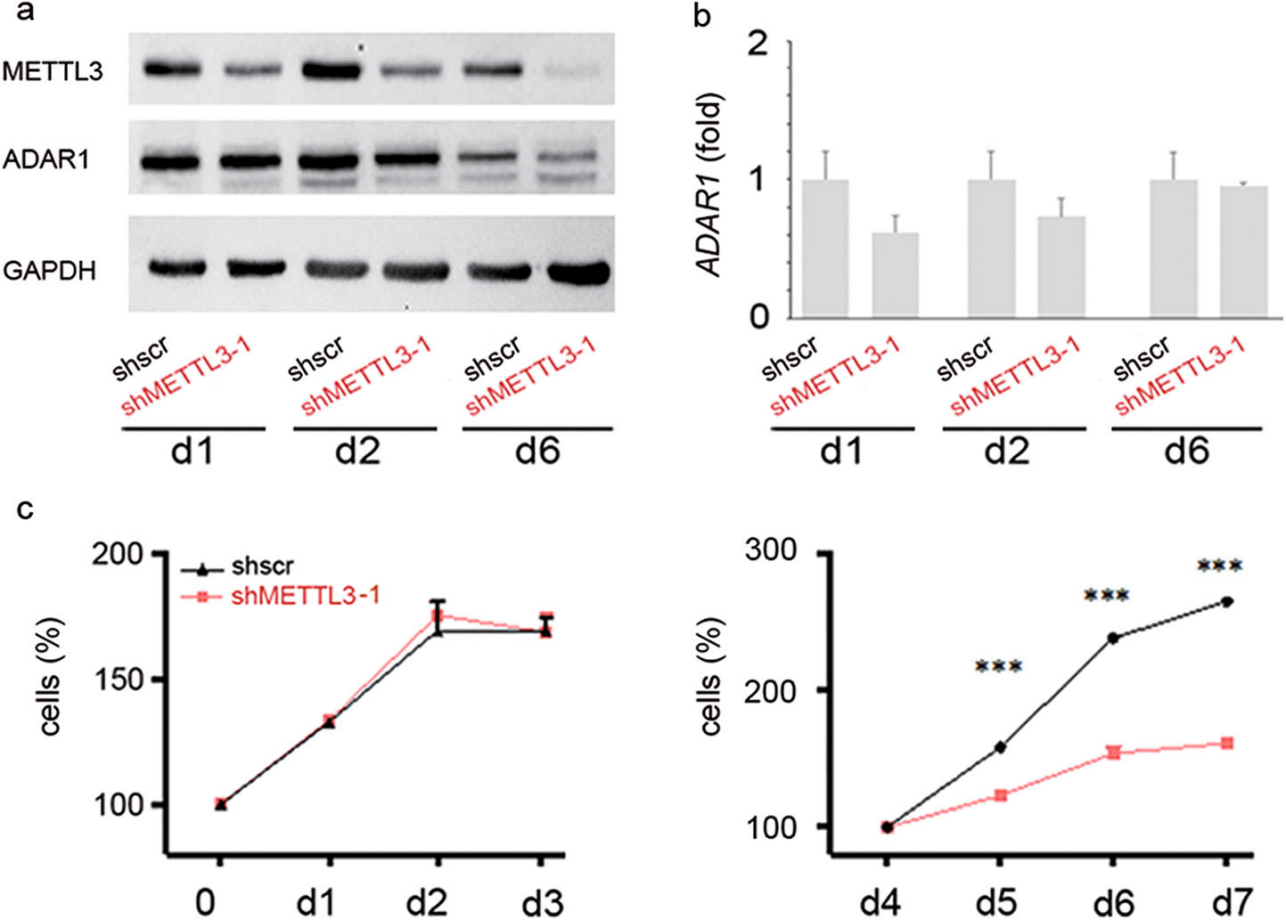
METTL3 silencing decreases ADAR1 protein level without affecting its mRNA and decreases cell proliferation after ADAR1 reduction. **a, b** Western blotting analysis and qRT-PCR of ADAR1 at day (d)1, d2, and d6 post doxycycline induction (shMETTL3–1) in U87MG cells. GAPDH was used as control. **c** Cell proliferation (MTS assay) of shMETTL3 U87MG cells compared to control cells at d1 to d7 post doxycycline (similar effect was observed in U118MG cells, see Additional file 2, Fig. S4). Values are represented as means ± SD, ***p ≤ 0.001

The m6A-modified RNA is recognized by specific proteins (readers) that transmit the “m6A-code” to downstream effectors within the cell. The m6A-binding proteins with YTH domain, including the cytoplasmic proteins YTHDF1, YTHDF2, YTHDF3, YTHDC2, and the nuclear protein YTHDC1, have been identified to be the “readers” of m6A and modulate mRNA stability and/or translation of target transcripts [14]. Interestingly, we observed that YTHDF1 was increased in GBM cells and tissues compared to controls (Fig. 3a and Additional file 1, Fig. S1b). Additionally, a recent study reported that, among the five YTH family members, the YTHDF1 was found to be the most highly upregulated in GBM samples (582 samples) compared with normal brain samples (720 samples) [15]. Therefore, to investigate whether YTHDF1 plays a role in m6A-regulated ADAR1 expression, we silenced this gene in glioblastoma cells, demonstrating that the knockdown of this “reader” significantly decreased ADAR1 protein, without affecting its mRNA (Fig. 3b). Therefore, we investigated whether YTHDF1 can bind *ADAR1* mRNA. YTHDF1-RIP experiments (performed with two different antibodies and internal controls) combined with qRT-PCRs were performed, demonstrating that YTHDF1 binds *ADAR1* mRNA (Fig. 4e).

In order to quantify the ribosome occupancy of ADAR1 mRNAs, mediated by its m6A modification, we performed ribosome immunoprecipitation experiments using U87MG human cells that expressed Flag-tagged RPL22 (ribosomal protein L22) upon YTHDF1 modulation [16]. Notably, we found that ribosomes accumulate ADAR1 mRNAs in control cells, while a reduced ADAR1 mRNA ribosome occupancy was observed in YTHDF1 silenced cells (Fig. 3d and Additional file 1, Fig. S3).

To determine whether the expression level of *ADAR1* is not correlated with changes in mRNA stability upon YTHDF1 modulation, we measured the levels of *ADAR1* mRNA after transcription inhibition with actinomycin D. We show that *ADAR1* mRNA stability was unaffected upon YTHDF1depletion (Fig. 3e). Therefore, to investigate whether protein decay can play a role in this scenario, we silenced YTHDF1 and treated the cells with or without MG132 (proteasome inhibitor), and then we measured ADAR1 protein level. ADAR2 [17] and anti-ubiquitin antibody were used as controls. We show that YTHDF1 can modulate ADAR1 protein independently of protein degradation mechanism (Fig. 3f).

Altogether, these data indicate METTL3/YTHDF1 as the post-transcriptional axis controlling ADAR1 protein levels by the translation modulation mechanism.

### Knockdown of METTL3 decreases ADAR1 protein levels together with cell proliferation

Considering the importance of m6A machinery in cell proliferation, we analyzed ADAR1 fluctuations and function upon METTL3 knockdown by using two different lentiviral vectors expressing doxycycline (dox)-inducible shRNA against METTL3 (shMETTL3 3–1 and shMETTL3 3–2) in two glioblastoma cell lines (U87MG and U118MG). A non-targeting scramble shRNA was utilized as control (shscr). We found that upon METTL3 downregulation, ADAR1 protein decreased independently of its mRNA (Fig. 4, Additional file 1, Fig. S4 and data not shown). Interestingly, monitoring cell proliferation of shscr and shMETTL3 cells over time, we found a progressive decrease in cell proliferation in shMETTL3 cells alongside with ADAR1 protein down expression (Fig. 4 and Additional file 1, Fig. S4). Indeed, while METTL3 is decreased already at day 1/2 post induction (Fig. 4 and Additional file 1, Fig. S4), cell proliferation decreased at day 5/6 and after ADAR1 protein reduction as observed in both cell lines and using two different lentiviral vectors expressing dox-inducible shRNA against METTL3 (Fig. 4, Additional file 1, Fig. S4 and data not shown).

Overall, our data indicate that shMETTL3 does not affect *ADAR1* mRNA levels, but directly decreases ADAR1 protein and METTL3-mediated cell proliferation changes occur after ADAR1 protein modulation.

### ADAR1 is the main target of *N*^*6*^-methyladenosine METTL3 controlling cell proliferation thanks to a mechanism independent of the deaminase activity

In order to investigate whether ADAR1 per se promotes cell proliferation in glioblastoma independently of METTL3, we silenced ADAR1 in several glioblastoma cell lines (U87MG, U118MG, A172, and T98G) and we tested cell proliferation over time. SiADAR1 cells decreased cell proliferation, thanks to the modulation of cell cycle at the G1/S transition without affecting cell apoptosis (Additional file 1, Fig. S5, S6). We found that ADAR1 downregulation strongly decreased the expression of CDK2 at mRNA and protein levels with ADAR1 binding *CDK2* mRNA, as shown by RNA immunoprecipitation experiments (Additional file 1, Fig. S5h).

Other important cell cycle proteins at the G1/S transition were tested, and among them we also analyzed proteins found to be modulated by ADAR2 (such as SKIP2 and CDC14B) [18], but none of these was found altered upon ADAR1 silencing (Additional file 1, Fig. S7).

In light of these findings, we investigated whether the pro-tumoral effect of METTL3 on cell proliferation (Fig. 4) is mainly mediated by ADAR1 and which ADAR1 isoform can be involved.

**Fig. 5:**
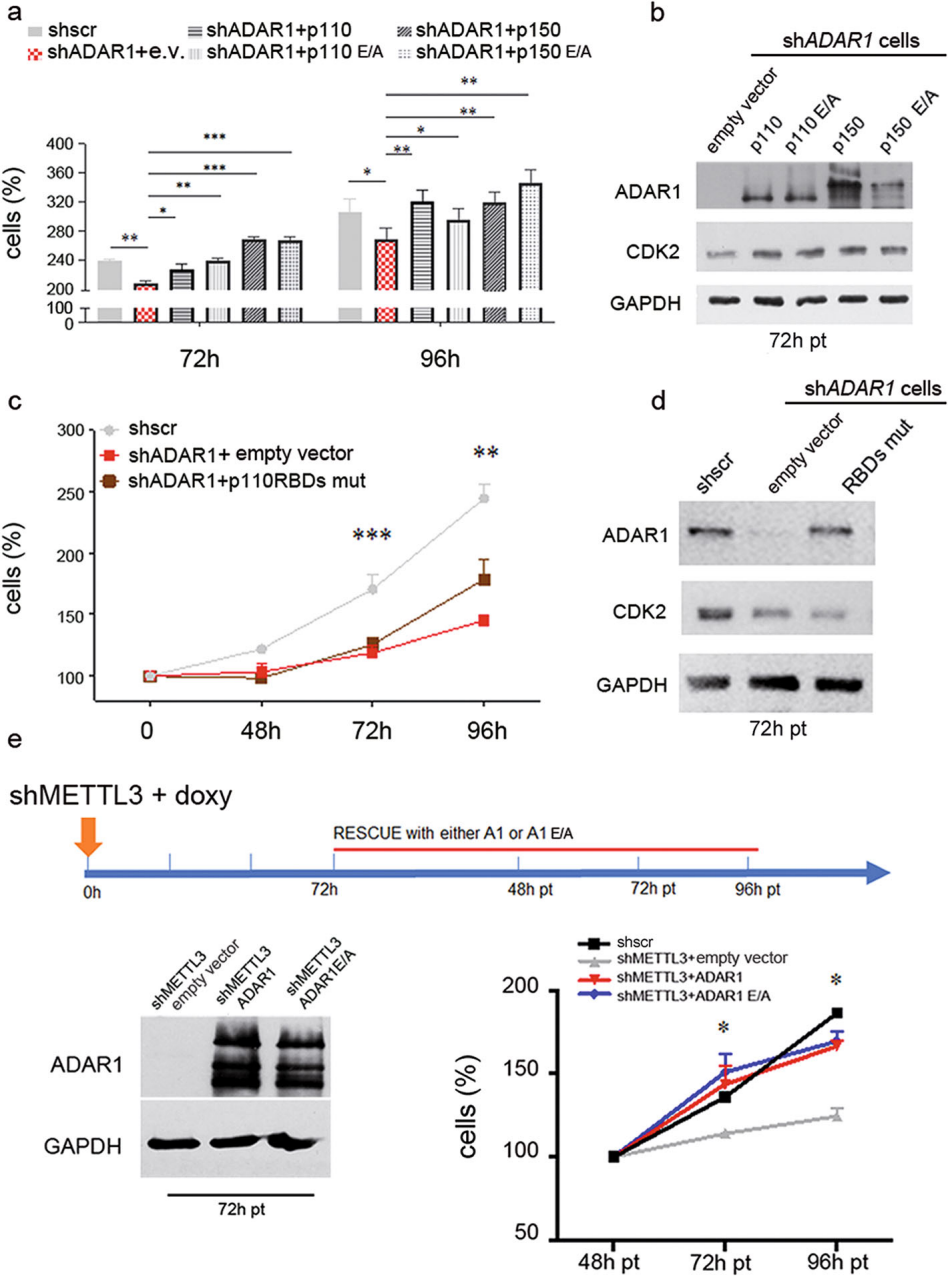
Both ADAR1 isoforms (p150 and p110), independently of their active deaminase domains, modulate CDK2 and rescue cell proliferation in shMETTL3 cells. **a** shADAR1 U87MG cells (with the shRNA targeting the 3′UTR of the endogenous *ADAR1*) were transfected with the active and the inactive (E/A) ADAR1 constructs (either p150 or p110) and cell proliferation (MTS assay) was tested at 72 and 96 h pt. **b** Western blotting analysis of ADAR1 and CDK2 proteins of the same cells at 72 h pt. are shown. **c**, **d** Cell proliferation (MTS assay) of shADAR1 U87MG cells transfected with ADAR1 p110 vector carrying an inactive RNA-binding domains (RBDs-mut) and western blotting analysis of ADAR1 and CDK2 proteins (72 h pt) is shown. **e** Cartoon showing the rescue experiment in shMETTL3 U87MG cells transfected with either ADAR1 (red), ADAR1 E/A (blue), empty vector (gray line), and untransfected cells (black line). On the left, a control western blotting analysis 72 h pt. showing that ADAR1 active and inactive were overexpressed at similar levels. GAPDH was used as control; on the right, the same cells were tested for cell proliferation (48–72 h pt). Values are represented as means ± SD, *p ≤ 0.05, **p ≤ 0.01, ***p ≤ 0.001

**Fig. 6:**
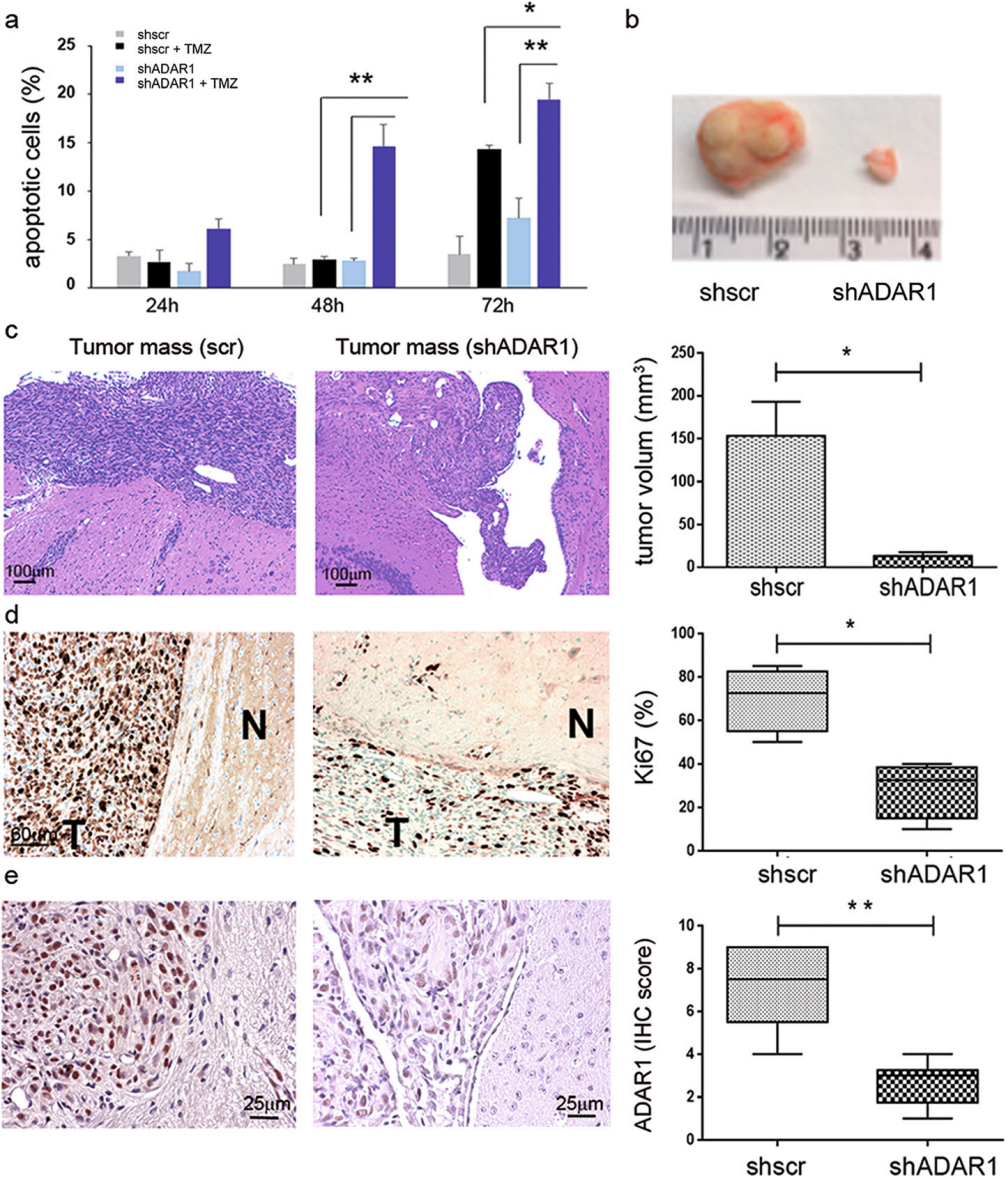
ADAR1 silencing sensitizes glioblastoma cells to TMZ treatment and inhibits glioblastoma growth in vivo. **a** shADAR1 U87MG cells were treated with or without 100 μM TMZ. Apoptotic cells were evaluated (Annexin V staining) 24–72 h post TMZ treatment. Values are represented as means ± SD, *p ≤ 0.05; **p ≤ 0.01. **b** An example of two similar experiments showing the difference of tumor mass (after 2 months) generated from shADAR1 and shscr U87MG cells subcutaneously injected into the flack of nude mice (*n* = 4 mice). **c** shADAR1 and shscr U87MG cells were injected intracranially (*n* = 6); a representative section (H&E staining) and tumor volume are shown, *p* = 0.0202 (Mann-Whitney *t*-test). **d** Left, representative section showing the Ki67 staining in brain tumors obtained in shADAR1 and shcsr U87MG cells. The tumor mass (T) and normal brain (N) are indicated. Right, Ki67 expression in the same tumors, *p* = 0.0286 (Mann-Whitney *t*-test). **e** Left, representative sections showing ADAR1 staining in brain tumors obtained with shADAR1 and shcsr U87MG cells. Right, ADAR1 expression in the same tumors, *p* = 0.0043 (Mann-Whitney *t*-test)

To this aim, we first investigated which ADAR1 isoform is important for cell proliferation by generating glioblastoma cells (U87MG) stably transfected with an inducible vector expressing a dox-inducible shRNA targeting the 3′UTR of *ADAR1*. Then, we performed serial rescue experiments in these cells with the endogenous *ADAR1* knocked down, re-introducing either p150 ADAR1 (active and the E/A catalytically inactive), p110 ADAR1 (active and the E/A catalytically inactive), or ADAR1 RNA-binding domain-mutated (RBDs-mut, unable to bind dsRNAs). Of note, as the in frame ATGs of the long ADAR1 transcript (starting from exon 1A [19]) can result in the translation of both ADAR1 p150 and p110, we have also utilized ADAR1 p150 vector carrying a point mutation (p150 GCG mut) within the second ATG (ATG to GCG), so that only the long p150 isoform can be generated. Cell proliferation and CDK2 levels were analyzed over days. We demonstrated that both ADAR1 isoforms (p110 and p150) are able to rescue cell proliferation and recover CDK2 expression (Fig. 5a, b). Identical cell proliferation rescue was also observed using the ADAR1 p150 and ADAR1 p150 GCG mut (data not shown). We also confirmed that shADAR1 decreases glioblastoma proliferation through CDK2 modulation, independently of its active deaminase domain as demonstrated by the p150 E/A and p110 E/A mutants (Fig. 6a, b). Additionally, the ADAR1 RBDs-mut, unable to bind dsRNAs, did not rescue cell proliferation and CDK2 levels (Fig. 5c, d).

Considering the role played by ADAR1 in controlling proliferation of glioblastoma cells, we wondered whether this protein could be responsible of the strong reduction of proliferation observed in shMETTL3 cells. Then, we performed an ADAR1 rescue experiment (ADAR1 p110 and ADAR1 p110 E/A) in shMETTL3 cells and we found that ADAR1, independently of its active deaminase domain, is sufficient to recover proliferation of glioblastoma shMETTL3 cells at a level similar to control levels (Fig. 5e), demonstrating that METTL3 mediates cell proliferation through ADAR1.

Overall, our data show ADAR1 as one of the main target of METTL3 controlling cell proliferation with both the ADAR1 isoforms (p110 and p150) able to modulate cell proliferation independently of their active deaminase domain, through the binding of *CDK2* mRNA.

### ADAR1 is a promising target for therapeutic intervention in glioblastoma

Glioblastoma is the most deadly type of brain cancer and is generally considered incurable. At present, the eligible chemotherapeutic treatment for glioblastoma includes temozolomide (TMZ), which is an alkylating agent that breaks the double-strand DNA, ultimately leading to cell death [20]. Unfortunately, due to its short half-life, TMZ is administered at high doses, and prolonged systemic administration has resulted in a series of side effects. In view of the data presented herein, in order to potentiate TMZ effects and reduce its negative consequences, we treated glioblastoma cells with a combined regime of shADAR1 (inhibition of cell proliferation) plus TMZ and monitored the cell death (apoptotic cells). U87MG cells stably transfected with shscr or shADAR1 were treated with TMZ (100 μM) and compared to the shscr cells (−dox). Annexin V staining was then performed at 24 h, 48 h, and 72 h post TMZ treatment. We report that ADAR1 down expression significantly boosts TMZ efficacy by (i) anticipating the TMZ effects and (ii) significantly increasing the amount of cancer cell death (Fig. 6a).

To translate the above results in an in vivo model, we tested the oncogenic potential of the shADAR1, with or without TMZ, in nude mice. Glioblastoma cells (U87MG) silenced for ADAR1 (shADAR1) and controls (shscr) were injected into the flanks of nude mice and tumor mass was monitored every 7 days for 2 months. No growing tumor mass was observed in shADAR1 cells compared to the controls that grew over time as expected (Fig. 6b). The combined treatment (with and without TMZ) was then not performed. Taking into consideration the above pilot experiment, we grafted the shADAR1 and control glioblastoma cells (U87MG) into the striatum of NOD-SCID mice (*n* = 6) and we monitored them over time. At 8 weeks after grafting, control mice harbored tumors that invaded the homolateral striatum, piriform cortex, corpus callosum, anterior commissure, internal capsule, optic tract, septal nuclei, and fimbria-hippocampus. On the contrary, shADAR1 glioblastoma cells did not develop an invading (spreading) tumor mass (Fig. 6c). The small masses developed by shADAR1 cells were also less proliferative compared to the mass originated from the control glioblastoma cells, as shown by Ki67 staining (Fig. 6d), and exhibited a significant decrease of ADAR1 and CDK2 protein expression (Fig. 6e and Additional file 1, Fig. S8).

**Fig. 7:**
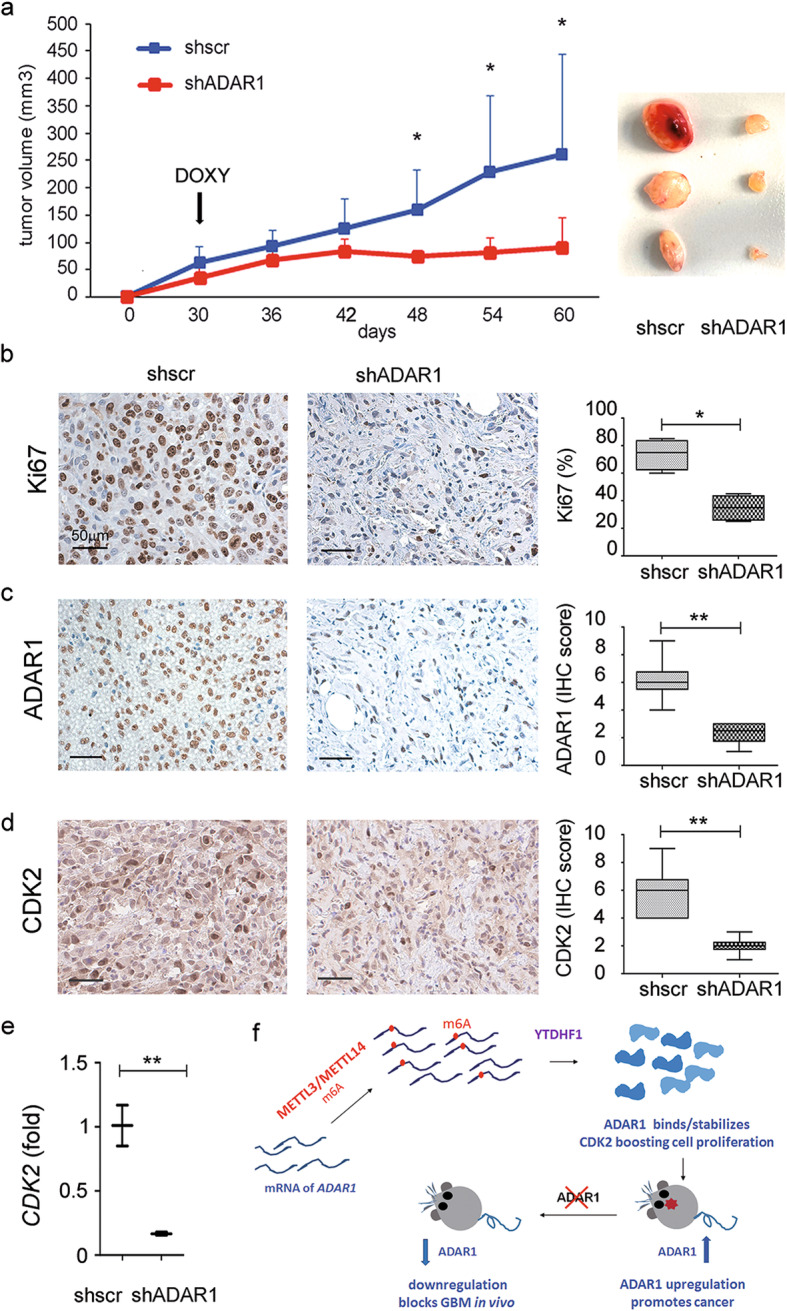
Targeting ADAR1 in growing tumor mass blocks glioblastoma progression*.* **a** Quantitative analysis of tumor size (tumor volume) of U87MG cells subcutaneously injected into the flank of NOD-SCID mice (*n* = 16 mice). Tumors generated by control (*n* = 8) or shADAR1 (*n* = 8) inducible U87MG cells were treated with DOXY (in drinking water). Tissues were collected and analyzed at the end of treatment (60 days p.i.). *p ≤ 0.05. Right, a representative picture of tumors. Representative sections and relative quantification of Ki67 (**b**), ADAR1 (**c**), and CDK2 (**d**) staining are shown. **e** qRT-PCR of *CDK2* using mRNA obtained from the same samples. Data were normalized to the mean of controls values set to 1. *p ≤ 0.05, **p ≤ 0.01. **f** Schematic representation of METTL3/ADAR1 modulation in glioblastoma. METTL3/METTL14 methylates *ADAR1* mRNA allowing the reader YTHDF1 to boost ADAR1 translation. The high level of ADAR1 protein (with unaltered ADAR1 mRNA) correlates with GBM patient OS and promotes cell proliferation by stabilizing CDK2. Moreover, the ablation of ADAR1 in an METTL3 unaltered background is sufficient to inhibit glioblastoma in vivo

Finally, in order to demonstrate that the ablation of ADAR1 when guided by external stimuli (such as lentivirus, compound, drug, DOXY, etc.) can inhibit tumor growth in vivo, we injected subcutaneously in the flanks of NOD-SCID mice (*n* = 16) the not induced glioblastoma cells (either shscr or shADAR1) and then, once the tumor mass started to growth, we treated the mice with DOXY (in drinking water) to induce, over time, the expression of shscr and the shADAR1 (Fig. 7a). The induction of ADAR1 silencing in an in vivo context totally blocks tumor growth for over 2 months (Fig. 7a and data not shown), differently from the control tumor that grows exponentially over time (Fig. 7a). The analysis of METLL3/METLL14 and YTHDF1 expression in these tumors is reported (Additional file 1, Fig. S9) together with that of Ki-67, ADAR1, and CDK2 that clearly indicates ADAR1 as an important target for glioblastoma and confirmed the modulation of the ADAR1/CDK2 axis in vivo (Fig. 7b–e).

Considering the strong effect of the induced shADAR1 on tumor growth and that the shADAR1 tumors, differently to the control mice, were really small and never growth over months (Fig. 7 and data not shown), the combined regime of shADAR1 with TMZ was unfeasible.

Overall, our data indicate ADAR1 as a promising therapeutic target for glioblastoma either in combination with TMZ (as indicated by in vitro studies) or alone (as demonstrated in vivo mouse model).

## Discussion

A-to-I and m6A are two of the most abundant modifications occurring at the RNA level both involving adenosines. While A-to-I RNA editing occurs in duplex structures of RNAs [21], m6A machinery largely involves single-stranded regions, at the RRACH motif, thanks to a set of writer and eraser proteins [22]. Hypothetically, the A sites for A-to-I or m6A can overlap; however, the different sequence and structure features for A-to-I or m6A suggest that these two chemical modifications do not likely compete for the same A bases. Indeed, m6A is not a good substrate for ADAR deamination at least in vitro assay [23]. Additionally, m6A can alter dsRNA structures important for the action of ADAR enzymes [24]. An intriguing and important question is whether m6A and A-to-I are totally independent mechanisms or they can interact somehow. A recent study demonstrated that m6A can alter the overall inosinome [25] probably through a perturbation of the RNA secondary structure necessary for the deamination [26]. Herein, we add a new important piece to the puzzle, showing that a direct link exists between these two post-transcriptional mechanisms with METTL3/METTL14/YTDHF1 able to boost ADAR1 protein levels.

In glioblastoma, we observed that both METTL3 and METTL14 are highly expressed compared to normal brain; additionally, we found that the A-to-I overall editing is decreased in GBM [10, 27], despite the high levels of ADAR1 protein found in this type of tumor (Fig. 1). How can all these data merge? It has been previously reported that a high level of METTL3 boosts the overall amount of transcripts carrying m6A, thus preventing editing at multiple sites [25] due to the unfavorable binding of ADARs to the m6A transcripts [26]. In this study, we demonstrate that METTL3 and YTHDF1 can directly target *ADAR1* transcript leading to a high level of its protein. All together, these findings indicate that ADAR1, despite being upregulated in GBM, due to the action of METTL3, might not be able to efficiently edit its targets probably due to the METTL3-dependent hyper-methylation of RNA substrates [28]. Indeed, in line with this hypothesis, our and other laboratories have showed that the *Alu*-editing index (AEI), mainly mediated by ADAR1, is decreased in glioblastoma compared to controls [10, 27].

The m6A is a reversibly process catalyzed by the methyltransferase complex and demethylated by the FTO and ALKBH5 erasers. We believe that, in different normal and cancer tissues, the balance between these two components (writers and erasers) can control ADAR1 protein level and the availability/editability of ADAR substrates. Our data revealed that *ADAR1* mRNA, its protein level, and the A-to-I editing are independently regulated and strongly connected with METTL3.

Indeed, our data showed that both METTL3 and YTHDF1 are able to control ADAR1 protein level. However, additional studies will be necessary to dissect whether a possible direct action of METTL3 over ADAR1 mRNA [29, 30] is possible or if other readers are involved.

The METTL3/YTHDF1 increases ADAR1 protein levels, leading to cell cycle acceleration. Indeed, we found that ADAR1, acting as an RNA-binding protein, binds and stabilizes the cyclin-dependent kinase 2 (*CDK2*) transcript. Of note, CDK2 is a key cell cycle kinase that promotes cell proliferation and is associated to a poor prognosis in multiple cancers [28].

METTL3 is also known to affect tumor formation/progression by the regulation of the m6A at critical oncogene or tumor suppressor transcript sites, and it is considered as a novel pharmacological target for the treatment of several cancers [31, 32]. Although the study of m6A is relatively young in glioblastoma, with the few studies on Glioma Stem Cells reporting divergent conclusions [13, 33], it is clear the key role played by m6A machinery also in glioblastoma. Indeed, a link between m6A and numerous cancer types has been reported; however, the impact of m6A on cancer cell proliferation is still under definition with more data still emerging. Our data indicate ADAR1 as one of the main targets of METTL3 controlling cell proliferation and connecting, in a new molecular pathway, two key proteins (METTL3 and CDK2) important for cancer progression.

We report that both ADAR1 p150 and ADAR1 p110, independently of their active deaminase domain, are able to boost cell proliferation increasing CDK2 level, demonstrating that these enzymes are also fundamental as RNA-binding proteins. ADAR proteins are linked to different types of cancers thanks to their ability to generate inosines (see as examples [11, 34, 35]); herein, we demonstrated that ADAR1, as an RNA-binding protein, is a powerful oncogene that inhibits cancer growth in vivo independently of its ability to generate inosines.

It has been recently shown that the catalytically inactive ADAR protein is able to rescue neurodegeneration phenotype in Adar mutants in *Drosophila*, suggesting that editing-independent roles of ADARs are involved in multiple pathways still little explored [36]; here, we show that ADAR are key proteins in cancer.

Glioblastoma may be one of the most challenging brain tumors to treat, as patients generally do not live more than 2 years. The treatment of this deadly cancer involves mainly the use of TMZ; however, prolonged and high dose of TZM generate negative side effects in patients. We found that a combined regime of shADAR1 and TMZ makes glioblastoma cells (in vitro) more sensitive to cell death. Surprisingly, we also found that targeting ADAR1 in glioblastoma tumor totally inhibits tumor growth in vivo. Altogether, our data pointed to ADAR1 as a key pro-tumoral protein that, if druggable, will represent a step forward in anticancer therapy.

Overall, our data disclose the novel molecular pathway METTL3/ADAR1/CDK2 connecting m6A and ADAR deaminases that can strongly change the scenario of post-transcriptional events with important consequences in cell homeostasis, differentiation, and cancer field.

## Conclusions

Herein, for the first time, we demonstrated that a direct link exists between METTL3 and ADAR1. Indeed, METTL3 increases ADAR1 protein level (independently of its mRNA). The high ADAR1 protein level correlates with glioblastoma patient survival and increases cell proliferation. We demonstrated that ADAR1 is one of the main targets of *N*^*6*^-methyladenosine METTL3 controlling cell proliferation in glioblastoma and its reduction in a METLL3-overexpressing background inhibits glioblastoma growth in vivo. Importantly, we demonstrated that ADAR1 acts as a pro-tumoral protein independently of its active deaminase domain. Several studies showed an important link between cancer and ADAR1 ability to generate inosine, thus pointing to the identification of molecules able to inhibit ADAR1 enzymatic activity as anticancer therapies. Our study indicates that, for an anti-cancer ADAR-mediated therapy, it could be more efficient to target ADAR1 protein than its deaminase activity.

## Materials and methods

### Human tissues and cell lines

De novo GBM tumors and control brain tissues (adult subjects) were dissected and either immediately frozen (for molecular studies) or embedded in *paraffin* (for immunohistochemistry analysis). Ethical approval for this study was obtained from Italian Ministry of Health.

This study includes 16 adult patients (11 men and 5 women) who underwent craniotomy for resection of histologically confirmed GBM (World Health Organization grade IV) in the supratentorial compartment and treated postoperatively with adjuvant radiotherapy and temozolomide (TMZ) at the Università Cattolica del Sacro Cuore (UCSC), Rome, Italy. The patients were aged 40 to 80 years at the time of primary surgery (median age, 58 years) [37].

The human glioblastoma cell lines U87MG, U118MG, T98G, and A172 obtained from American Type Culture Collection (ATCC) were routinely maintained in Dulbecco’s modified Eagle’s medium (DMEM) supplemented with 10% fetal bovine serum (Gibco-Life Technologies), 100 U/ml penicillin, and 100 μg/ml streptomycin, at 37 °C in 5% CO^2^. Human primary astrocytes (Euroclone) were maintained for few passages in ABMTM astrocyte cell basal medium following the manufacturer’s instructions. Doxycycline (dox), temozolomide (TMZ), MG132, and actinomycin D were purchased from SIGMA.

### MG132 and actinomycin D treatments

To inhibit proteasome, U87MG cells were incubated with MG132 (SIGMA) for 24 h. Different doses of MG132 were used as described in the text. Proteasome inhibition was performed using 1.25 μM MG132 in control and siYTHDF1 U87MG cells 24 h post transfection. Cells were harvested 24 h later then total RNA and protein extracts were isolated to perform qRT-PCR and western blotting analysis (see next sections).

To measure RNA stability, 5 μg/ml actinomycin D (SIGMA) was added to cells for 10 h. Both RNA and protein extraction was performed as described in the next sections.

### Transfected stable cell lines

Stable shADAR1 glioblastoma cells (U87MG, U118MG, T98G, and A172) were generated using SMARTvector Inducible Lentiviral shRNA according to the manufacturer’s instructions (Dharmacon). Cells were selected with 1 μg/mL puromycin (starting from 2 days post infection) and expanded. Induction of shRNA was obtained using 1 μg/mL doxycycline that was refreshed every 2 days. For METTL3 downregulation in U87MG and U118MG glioblastoma cells, an inducible shRNA expression system based on the lentiviral vector pLKO-Tet-On was used, as described in Sorci and co-authors [38]. Cells were selected with 1 μg/mL puromycin (starting from 2 days post infection) and expanded. Induction of shRNA was obtained using 100 ng/mL doxycycline that was refreshed every 2 days.

### Transient cell line transfections

For ADAR1 rescue experiments, inducible shADAR1 U87MG cells were treated with 1 μg/ml doxycycline for 3 days to silence ADAR1, then transfected with plasmids containing both ADAR1 isoforms (p110 and p150) in the active and inactive (E/A) form. Transfection was performed using Lipofectamine 2000 (Invitrogen-Life Technologies) following manufacturer’s instructions.

Si*YTHDF1* and control siRNA were purchased from QIAGEN, while the siADAR1 and control were purchased from Eurofins (sequences can be provided under request). Specifically, a total of 100 nM siRNA was transfected using Oligofectamine (Invitrogen-Life Technologies), according to the manufacturer’s instructions.

### Site direct mutagenesis

ADAR1 p150 and ADAR1 p110 pEXFH plasmid vectors were kindly provided by Stefan Mass and previously described [39]. Catalytically inactive pEXFH ADAR1 p150 and ADAR1 p110 were generated by changing a key glutamate residue in the deaminase domain with alanine (E/A). Site direct mutagenesis was performed with the following primers: FW 5′-TCAATGACTGCCATGCAGCAATAATCTCCCGGAGAGG-3′ and REV 5′- CCTCTCCGGGAGATTATTGCTGCATGGCAGTCATTGA-3′ using the QuickChange® kit (Agilent) according to the manufacturer’s instructions. A second mutagenesis was performed in the pEXFH ADAR1-p150 and pEXFH ADAR1-p150 E/A changing the methionine located in exon 2 from which the transcription of the shorter ADAR1 isoform starts (ADAR1 p110). Site direct mutagenesis was performed changing methionine with alanine with the following primers: FW 5′- CTCTTGAGTTTTTAGACGCGGCCGAGATCAAGGAGAAAATC-3′ and Rev. 5′- CTCTTGAGTTTTTAGACGCGGCCGAGATCAAGGAGAAAATC-3′ using the QuickChange® kit (Agilent) according to the manufacturer’s instructions. The pCD3 plasmid vector containing the ADAR1 p110 construct with the three RNA-binding domains mutated was kindly provided by Kazuko Nishikura [40].

### RNA isolation and qRT-PCR

Total RNA fractions were isolated using TRIzol reagent (Invitrogen-Life Technologies) according to the manufacturer’s instructions. RNA concentration and purity (A260/A280 nm ratio) were evaluated using a NanoDrop ND-2000 (Thermo Scientific). Total RNA was treated with DNase I (Ambion) and reverse transcribed using the ImProm-II Reverse Transcription System (Promega). Quantitative real-time polymerase chain reaction (qRT-PCR) was performed to validate the expression of specific mRNAs, using pre-designed assays (TaqMan Applied Biosystems-Life Technologies). *β-actin* or *GAPDH* were used as control for mRNA normalization. The relative amount of each substrate was calculated by the 2^−ΔΔCt^ method. Expression levels were represented as relative fold increase compared to the control sample, which was arbitrarily set to 1. All qRT-PCR reactions were performed in duplicate or triplicate and repeated at least twice from independent RT-PCRs, *p* values were calculated (two-sided *t*-test), and values are represented as mean ± SD. RNA isolated during m6A immunoprecipitation experiments was quantified using the SYBR green dye detection system, which was performed in duplicate. Relative expression levels of targets were determined using the comparative 2^−∆∆Ct^ method. Specifically in these experiments, the oligos used were:

*ADAR1* Fw_1 5′-GCCAAGAAAGCTGCCCGT-3′, Rev_1 5′-CAGTTCCCATAGCCCATATCCT-3′; Fw_2 5′- GGAACTGGATTAGCAAACCCC-3′, Rev. 5′- ACCCTAATCCATCTGTCACTGG-3′; *HPRT* Fw 5′- TGTCAGTTGCTGCATTCCTA − 3′, Rev. 5′-ACCCTAATCCATCTGTCACTGG-3′.

All the reactions were performed on an Applied Biosystems 7500 Fast Real Time PCR System.

### m6A immunoprecipitation

m6A immunoprecipitation was performed as described by Dominissini and co-authors [41]. Briefly, total RNA from U87MG, U118MG, and shMETTL3 U87MG cells was extracted and fragmented into ~ 100-nt-long fragments in Fragmentation Buffer (100 mM Tris–HCl and 100 mM ZnCl2) for 5′ at 94 °C. Reaction was immediately blocked with addition of EDTA 50 mM. A portion of fragmented RNA was kept as input control, while 50 μg of fragmented RNA was immunoprecipitated in 1 ml of IP Buffer (50 mM Tris–HCl, 750 mM NaCl, and 0.5% Igepal CA-630) complemented with RNasin (400 U), with 2 μg of m6A-specific antibody (ab151230, Abcam) or 2 μg of control rabbit IgG (Millipore) for 2 h of incubation at 4 °C on rotator. Then, 20 μl of protein A beads (Invitrogen), saturated with BSA (SIGMA) 0.5 μg/ml for 2 h was added and the reaction mixtures and incubated for 2 h at 4 °C on rotator. After incubation beads were spinned down and washed three times with IP Buffer. Elution was performed incubating the beads four times in Elution Buffer (150 mM NaCl, 50 mM Tris–HCl pH 7.5, 1 mM EDTA, 0.1% SDS, 20 mM DTT) for 5′ at 42 °C. Eluted RNA was precipitated with addition of one-tenth volumes of 3M sodium acetate (pH 5.2) and 2.5 volumes of 100% ethanol and incubated overnight at − 80 °C. Precipitated RNA was then centrifuged at 15,000*g* for 25′ at 4 °C and pellet resuspended in 15 μl of RNase-free water.

### Ribosomal immunoprecipitation

To perform ribosome immunoprecipitation, a RPL22-Flag construct (Origene, kindly provided by Prof. A. Fatica) was transfected in control and si*YTHDF1* U87MG cells 48 h post siRNA treatment using Lipofectamine 2000 (Invitrogen-Life Technologies) following the manufacturer’s instructions. Two days later, the same amount of protein lysates from sicontrol and si*YTHDF1* U87MG cells was incubated O/N with 5 μg of anti-FLAG antibody (SIGMA) or normal mouse immunoglobulin G (IgG) as control. RNA immunoprecipitation was performed as described in the next section.

### RNA immunoprecipitation (RIP)

Total RNA immunoprecipitation was performed with the EZ-Magna RIP™ RNA Binding Protein Immunoprecipitation Kit (Merk) following the manufacturer’s instructions. Immunoprecipitation were carried out using 5 μg of anti-ADAR1 antibody (Bethyl), anti-YTHDF1 (Abcam, Ab220162 and Ab99080), anti-FLAG (SIGMA), or normal rabbit/mouse immunoglobulin G (IgG) as control and incubating at 4 °C overnight. Precipitated RNA was then centrifuged at 15,000*g* for 30′ at 4 °C and pellet resuspended in 15 μl of RNase-free water.

### Immunoblotting

Total protein extracts were isolated with RIPA lysis buffer in the presence of a protease inhibitor mixture and phosphatase inhibitor cocktail (SIGMA). Protein extracts were quantified with the BCA Protein Assay Kit (Pierce). Equal amounts of total cellular lysates (30 μg) were separated by sodium dodecyl sulphate-polyacrylamide gel electrophoresis (SDS-PAGE), transferred to nitrocellulose membrane, analyzed by immunoblotting with the appropriate antibodies and then revealed by ECL (GE Healthcare). The antibodies used were as follows: ADAR1 (Santa Cruz Biotechnology), ADAR1 (Bethyl), CDK2 (Santa Cruz Biotechnology), YTHDF1 (Abcam), METTL3 (Abcam), METTL14 (Bethyl), cyclinE (Santa Cruz Biotechnology), p57 (Santa Cruz Biotechnology), Skp2 (Santa Cruz Biotechnology), CDC14B (LifeSpan), ADAR2 (Santa Cruz Biotechnology), Ubiquitin (Thermo Fisher), β-actin (Santa Cruz Biotechnology), GAPDH (Cell Signaling), and the anti-rabbit and anti-mouse peroxidase-conjugated secondary antibodies (Santa Cruz Biotechnology).

### Immunofluorescence and confocal microscopy analysis

U87MG and U118MG glioblastoma cell lines were adhered onto poly-L-lysine glass slides and fixed for 10 min at room temperature in 4% paraformaldehyde. Cells were washed twice in PBS, permeabilized for 10 min with PBS containing 0.1% Triton-X-100 and incubated for 1 h with PBS containing 0.5% BSA. Cells were then incubated o/n at 4 °C with anti-ADAR1 antibody (Bethyl) which was diluted 1:500 and anti-METTL3 antibody (Abcam) which was diluted 1:200 and anti-METTL14 antibody (Bethyl) diluted 1:100. After washing, secondary antibody (alexa Fluor 488 Invitrogen) was added for 1 h at room temperature. Slides were washed and mounted in 50% glycerol in PBS. Hoechst 33342 (Sigma Aldrich) was used to counterstain nuclei. Confocal imaging was performed on an Olympus Fluoview FV1000 confocal microscope equipped with FV10-ASW version 4.1a software.

### Cell proliferation

Cells were seeded and transfected in 6-well dishes. Viability (Trypan blue dye exclusion) was determined daily, from day 1 to day 4 post siRNA transfection. The assay was repeated at least three times in duplicate. For statistical analysis, we used the two-sided *t*-test.

### MTS assay

Cell proliferation was evaluated by tetrazolium compound MTS [3-(4,5-dimethylthiazol-2-yl)-5-(3-carboxymethoxyphenyl) 2-(4-sulphophenyl)-2H–tetrazolium, inner salt] (Promega). Briefly, cells were plated in triplicate in 96-well plates and 20 μl of MTS solution was added to each well every day and cells were incubated at 37 °C for 2 h. Absorbance intensity was determined on a microplate reader at 490 nm. The assay was repeated three times in triplicate. Proliferation was measured as fold increase over 0 h.

### Cell cycle and apoptosis analysis

For cell cycle analysis, 2 × 10^5^ glioblastoma cells were resuspended in PBS with 200 mg/mL propidium iodide (PI) and 200 mg/mL RNAse A or tested by Click-iT (Thermo Fisher) following the manufacturer’s instructions. Cells were analyzed on a FACSCanto II flow cytometer (Beckton Dickinson) using the DIVA software. Apoptosis was detected by using the Annexin V-FITC apoptosis detection kit I (BD Pharmingen). Briefly, cells attached to the plate as well as those present in the supernatant were collected together and resuspended in 1× binding buffer at a concentration of 1 × 10^6^ cells per ml. A 200 μl sample of solution containing 2 × 10^5^cells was incubated with 10 μl of AnnexinV-FITC and 10 μl of 7-aminoactinomycin D for 15 min at room temperature in the dark, followed by addition of 200 μl of one time binding buffer. Samples were then analyzed on a FACSCanto II flow cytometer (Beckton Dickinson) using the DIVA software. Apoptotic cells, staining positive for Annexin V-FITC and negative for 7-aminoactinomycin D, were counted and represented as a percentage of the total cell count.

### In vivo experiments

2 × 106 U87MG cells already expressing shscr or shADAR1 were subcutaneously injected in the flank of 6-week-old nude mice (nu/nu, Charles River, Wilmington, MA, USA).

Additionally, a total of 16 mice were subcutaneously injected into the flank of CD1 NOD−/SCID mice (Charles Rives, Italy, with 8 mice carrying shADAR1 and 8 mice shcrl), and only after 30 days post injection, the doxycycline was administrated in drinking water (200 μg/ml) when the tumor started to grow. Tumor size was assessed by caliper measurement. Tumor volume was calculated as follows: volume (mm^3^) *D* × *d*^2^ × π/6, where *D* and *d* are the longest and the shortest diameters, respectively.

For orthotopic mouse models, 2 × 10^5^ shscr and shADAR1 U87MG glioblastoma cells were intracranially injected into male NOD/SCID mice (*n, 6*; 6–8 weeks of age; CD1 NOD−/SCID mice, Charles Rives, Italy). Before grafting, mice were anesthetized with intraperitoneal injection of xilazina (10 mg/kg) followed by intramuscular injection of ketamine (200 mg/kg). The animal skulls were immobilized in a stereotactic head frame and a burr hole was made 2 mm right of the midline and 1 mm posterior to the coronal suture. The tip of a 10-μl Hamilton microsyringe was placed at a depth of 3 mm from the dura, and the cells, resupended in 5 μl of PBS, were slowly injected with a flow rate 0.5 μl/min. Doxycycline administration in drinking water (200 μg/ml) started the day of injection. After 8 weeks of survival, mice were deeply anesthetized and transcardially perfused with 0.1 M PBS (pH = 7.4), followed by 4% paraformaldehyde in 0.1 M PBS. The brain was removed and paraffin fixed for morphological and immunohistochemical analysis. The extension of the brain area invaded by U87MG cells was assessed on serial sagittal sections. To assess the tumor volume, each area of the infiltrated brain was multiplied for the distance to the consecutive digitized section, starting from the tumor epicenter to the cranial and caudal poles of the tumor, and partial volume values were added. Alternate sections were stained with hematoxylin and eosin (H&E) for morphological analysis. Ethical approval for this study was obtained from Italian Ministry of Health.

### Immunohistochemistry

For immunohistochemistry analysis and H&E staining, formalin-fixed, paraffin-embedded sections (3 μm thick) were mounted on positively charged glass slides. Deparaffinization and antigen retrieval was performed using the PT link instrument (Dako) and the EnVisionTM FLEX, low pH solution (Dako). Endogenous peroxidase was blocked by hydrogen peroxide (SIGMA), then sections were incubated at 4 °C overnight with mouse monoclonal antibody anti-ADAR1 1:100 dilution (Santa Cruz Biotechnology), rabbit monoclonal antibody anti-METTL3 1:100 dilution (Abcam), anti-CDK2 1:200 dilution (Santa Cruz Biotechnology), rabbit polyclonal antibody anti-Ki67 1:100 dilution (Abcam), rabbit polyclonal antibody anti-METTL14 1:100 dilution (Bethyl), rabbit polyclonal antibody anti-YTHDF1 1:100 dilution (Abcam), mouse monoclonal antibody anti-GFAP 1:100 dilution (cell signaling), and rabbit monoclonal antibody anti-synaptophysin 1:100 dilution (Abcam) followed by EnVision FLEX/HRP (Dako). 3,3′ Diaminobenzidine was used as the enzyme substrate to observe the specific antibody localization and Mayer hematoxylin was used as a nuclear counterstain. H&E staining was performed following standard procedures. The staining intensity of tissue slides was evaluated independently by 2 observers (V.C. and M.M.) who were blinded toward the patients’ characteristics and survival. Cases with disagreement were discussed using a multiheaded microscope until agreement was achieved. To assess differences in staining intensity, an immunoreactivity scoring system was applied. ADAR1 and CDK2 expression in each specimen was scored according to the extent (percent of stained cells) and intensity of nuclear expression staining, at least 250 cells in each sample were analyzed. The score for the percentage of stained cells was scaled as 0 for no IHC signal at all, 1 for 1–30%, 2 for 31–70%, and 3 for 71–100% of tumor cells stained. The score for IHC intensity was scaled as 0 for no IHC signal, 1 for weak, 2 for moderate, and 3 for strong IHC signals. The final score used in the analysis was calculated by multiplying the extent score and intensity score, with a maximum score equal to 9. Immunohistochemical score between 0 and 3 was defined as low protein expression level while score from 4 to 9 was defined as high protein expression level [42]. Negative controls were tumor sections stained in the absence of the primary antibody. Positive controls were human nasopharynx tissue (showing nuclear staining of respiratory epithelial cells) samples for ADAR1. Ki67 index was evaluated as the percentage of positive nuclear staining cancer cells. All samples were stained more than once, and the results were highly reproducible.

### Statistical analysis

The statistical analyses were performed using the two-tailed Student’s *t* test and values are represented as means ± SD and statistical significance was set at *p* ≤ 0.05. Kaplan-Meier survival curves were plotted and differences in survival between groups of patients were compared using the log-rank test and Gehan-Breslow-Wilcoxon performed by GraphPad Prism 7 software. Overall survival (OS) was calculated from the date of surgery to death or end of follow-up. For the analysis of the brain tumor volume (mm^3^) and Ki67 (%), the statistical significance was calculated with the Mann-Whitney *t*-test performed by the GraphPad Prism 7 software. Experiments were repeated independently multiple times and similar results were obtained.

## Supplementary Information

**Additional file 1: Supplementary Figs. S1-S6** plus figure legends.

**Additional file 2: Table S1.** Patients characteristics.

**Additional file 3.** Uncropped western blotting analysis.

**Additional file 4.** Review history.

## Data Availability

Glioma stem cell m6A RIP-seq were downloaded from SRP099397 repository https://www.ncbi.nlm.nih.gov/sra [13].

## Abbreviations

ADAR: Adenosine deaminases acting on dsRNA
METTL3: Methyltransferase-like 3
GBM: Glioblastoma
METTL14: Methyltransferase-like 14
MTC: m6A methyltransferase complex
WTAP: Wilms tumor 1-associated protein
YTHDF1: YTH domain-containing family protein 1
YTHDF2: YTH domain-containing family protein 2
YTHDF3: YTH domain-containing family protein 3
YTHDC2: YTH domain-containing proteins 2
YTHDC1: YTH domain-containing proteins 1
CDK2: Cyclin-dependent kinase 2
TMZ: Temozolomide
dox: Doxycycline
RIP: RNA immunoprecipitation
RBD: RNA-binding domain
GFAP: Glial fibrillary acidic protein
MTS-3: (4,5-Dimethylthiazol-2-yl)-5-(3-carboxymethoxyphenyl)-2-(4-sulfophenyl)-2H-tetrazolium)
