# Supplementary material for: ADAR1 is a new target of METTL3 and plays a pro-oncogenic role in glioblastoma by an editing-independent mechanism
Source: Genome Biol. 2021 Jan 28;22:51. doi: 10.1186/s13059-021-02271-9 (PMC7842030; doi:10.1186/s13059-021-02271-9)
Supplement: Supplementary file 1 — Additional file 1: Supplementary Figs. S1-S6 plus figure legends. [file 13059_2021_2271_MOESM1_ESM.pdf]

Supplementary Information

Additional File 1

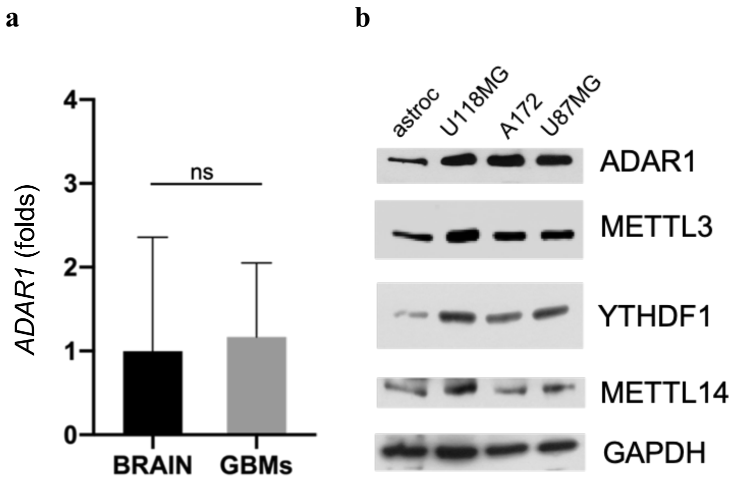

Fig. S1. ADAR1, METTL3, METTL14 and YTHDF1 expression in astrocytes, GBM cell lines and GBM tissues. a) qRT-PCR of *ADAR1* mRNA level in normal brain cortex (n=2) and primary adult GBM tissues (n=8). Data were normalized to the mean of controls values set to 1. **b)** Western blotting analysis of ADAR1, METTL3, METTL14 and YTHDF1 in primary astrocytes and GBM cell lines.

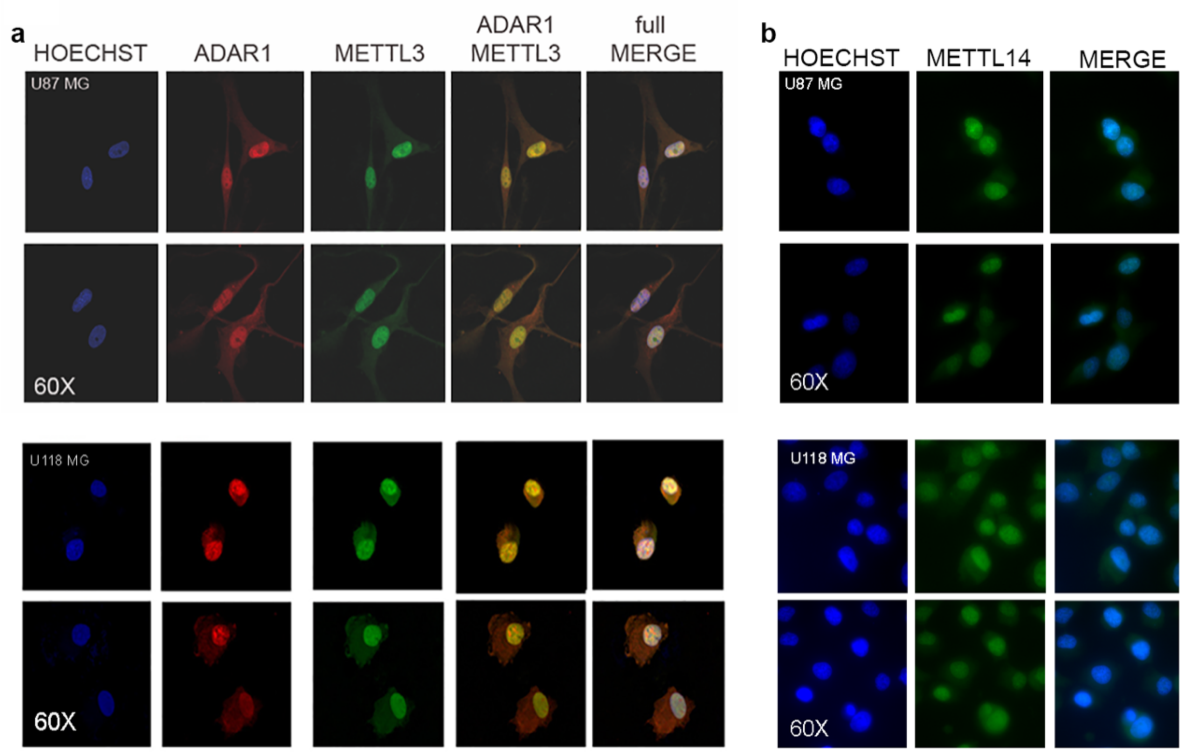

Fig. S2. Immunolocalization of ADAR1, METTL3 and METTL14 in U87MG and U118MG cells. Immunofluorescence showing ADAR1 (red), METTL3 (green) **(a)** and METTL14 (green) **(b)** in U87MG and U118MG cells. Nuclei were counterstained with HOECHST (blue color). Merged images are shown.

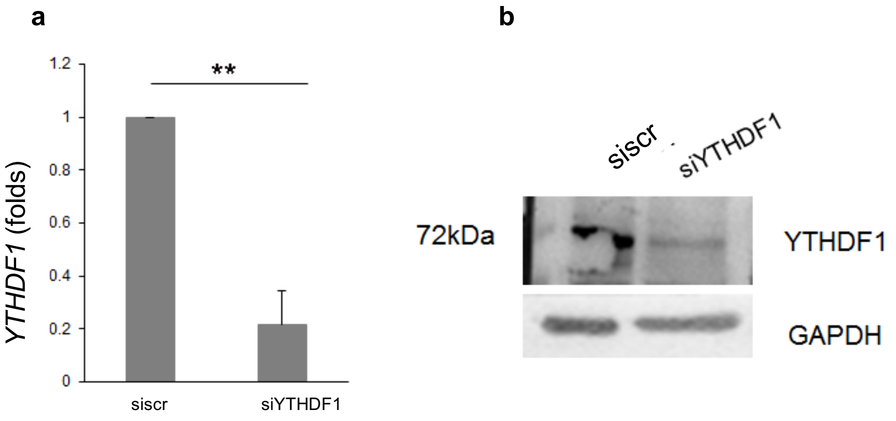

Fig. S3. YTHDF1 silencing in RPL22 transfected cells. a) qRT-PCR and **b)** western blotting analysis of control and siYTHDF1 U87MG cells transfected with a flag-tagged RPL22 construct (ribosomal protein L22). Values are represented as means ± SD, $^{**}p \leq 0.01$.

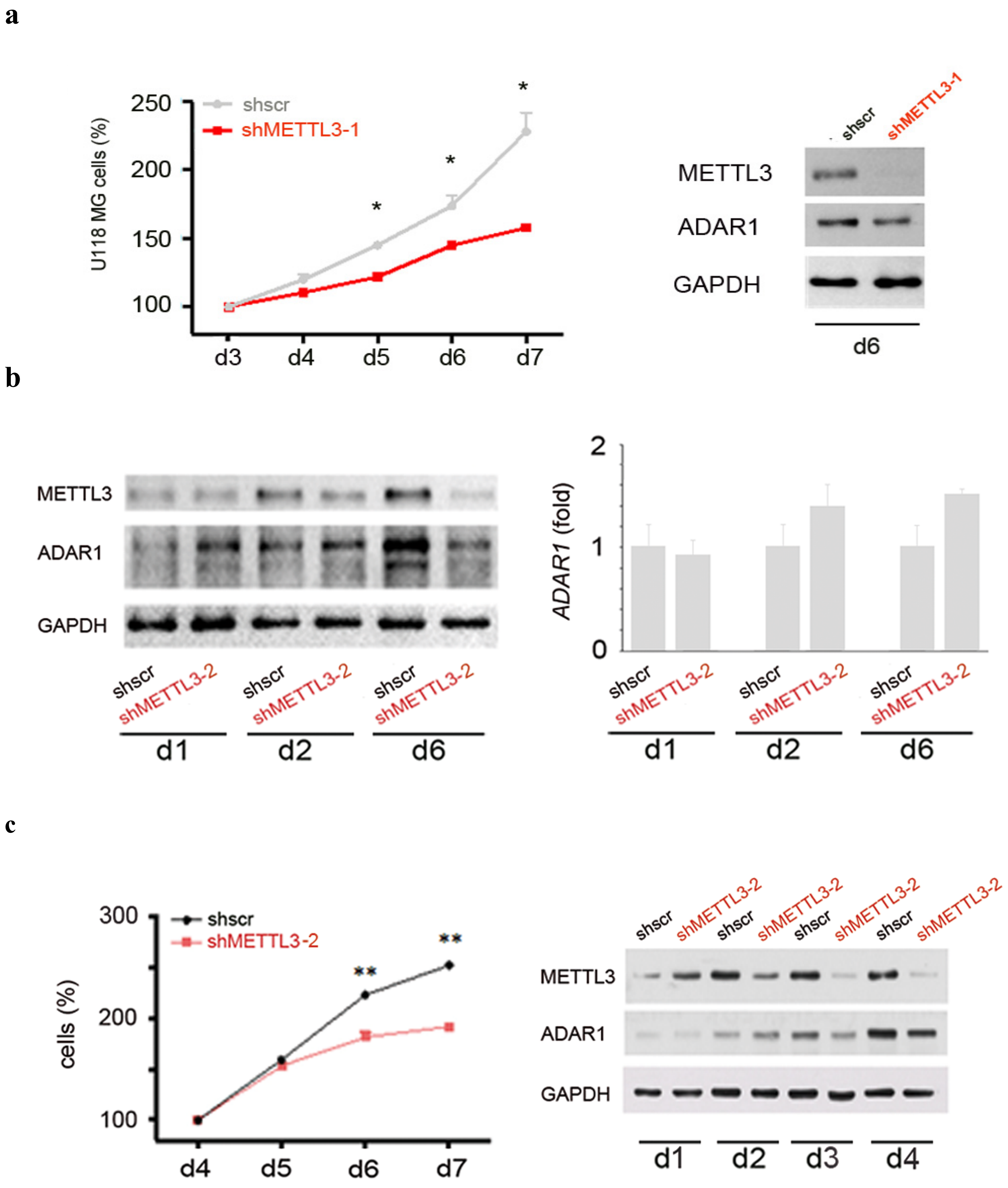

Fig. S4. METTL3 silencing decreases cell proliferation and ADAR1 protein level. a Cell proliferation (MTS assay) of shMETTL3-1 U118MG cells from d3 to d7 post doxycycline induction. On the right, western blot showing ADAR1 and METTL3 at d6 post doxycycline in U118MG shMETTL3-1 cells. GAPDH was used as a control. **p* value ≤ 0.05. **b** Western blotting analysis and qRT-PCR of ADAR1 at day (d)1, d2 and d6 post doxycycline induction in U87MG cells (using a different lentiviral construct, shMETTL3-2). **c** Cell proliferation (MTS assay) is shown from d4-d7. On the right, western blot showing ADAR1 and METTL3 at d1-4 post doxycycline in. GAPDH was used as a control. Values are represented as means ± SD, ***p* ≤ 0.01.

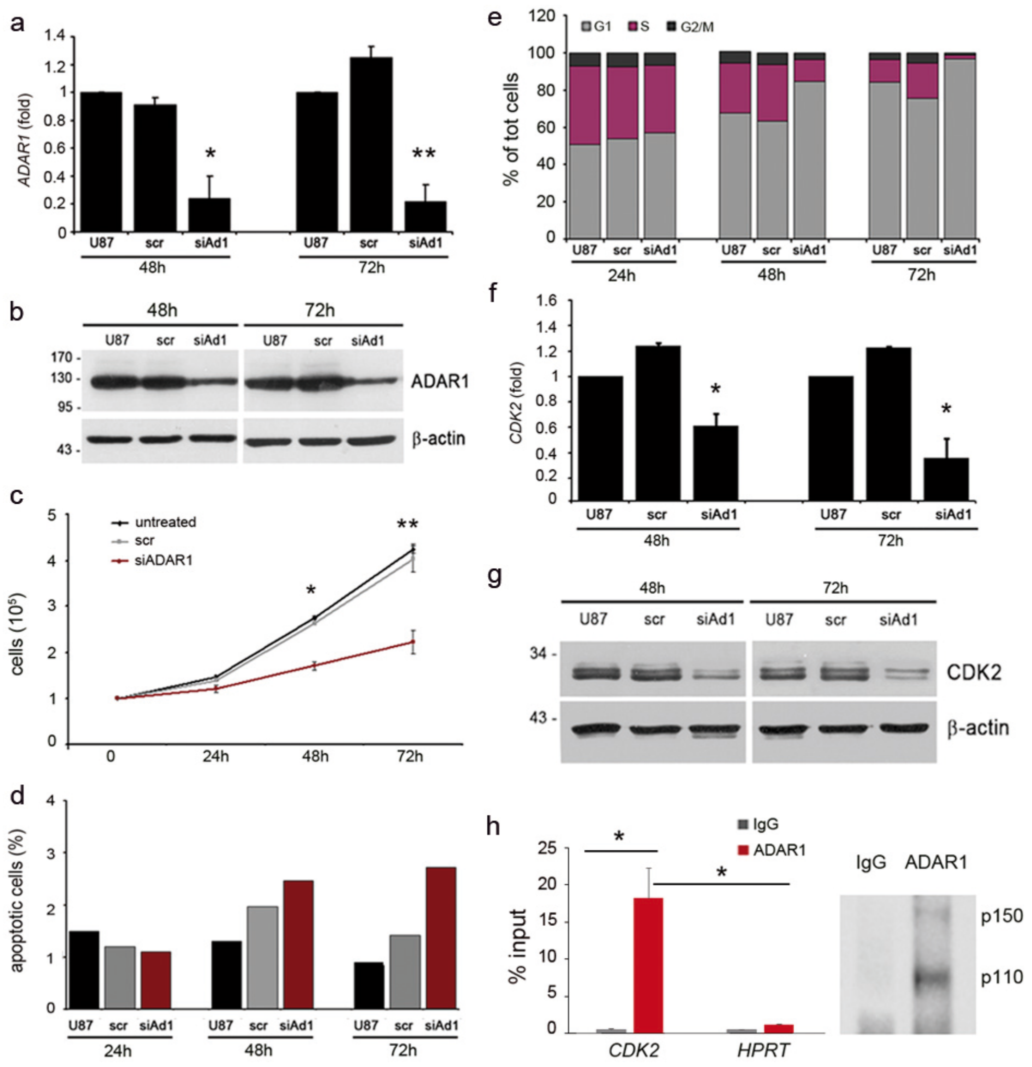

Fig. S5. ADAR1 controls the cell cycle at G1/S transition. (a, b) QRT-PCR and western blot analysis of ADAR1 in siADAR1 (siAd1), sicontrol (scr) and untreated U87MG cells. (c-e) Cell proliferation, apoptosis and cell cycle analysis of siADAR1 (siAd1), control (scr) and untreated U87MG cells performed simultaneously. (f, g) QRT-PCR and western blot analysis of CDK2 in siADAR1 (siAd1), control (scr) and untreated U87MG cells, β-actin was used as a control. (h) Relative enrichment of *CDK2* mRNA in ADAR1-RIP over IgG in U87MG cells, *HPRT* expression was used as negative control. A western blot analysis of RIP experiments is shown. Values are represented as means ± SD, *$p \leq 0.05$, **$p \leq 0.01$.

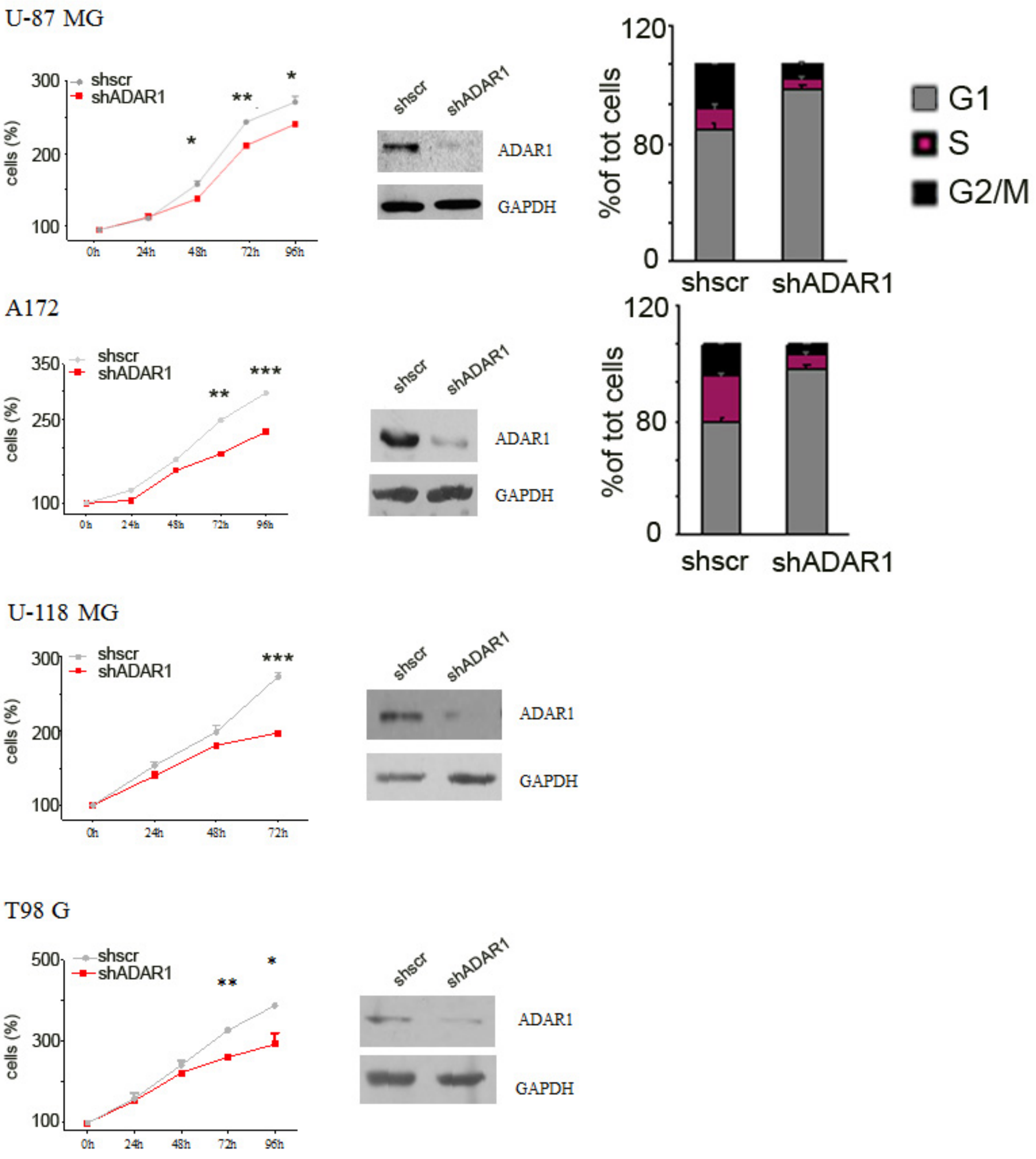

Fig. S6. ADAR1 silencing significantly decreases cell proliferation in different glioblastoma cell lines. Cell proliferation (MTS assay) of doxycycline inducible shADAR1 and shscr U87MG, A172, U118MG and T98G glioblastoma cells. ADAR1 protein expression was evaluated by western blot analysis at 72h post doxycycline induction and GAPDH was used as control.* *p* value ≤ 0.05, ***p* value ≤ 0.01, ****p* value ≤ 0.001. Cell cycle analysis (% of total cells) is shown for shADAR1 U87MG and A172 glioblastoma cells.

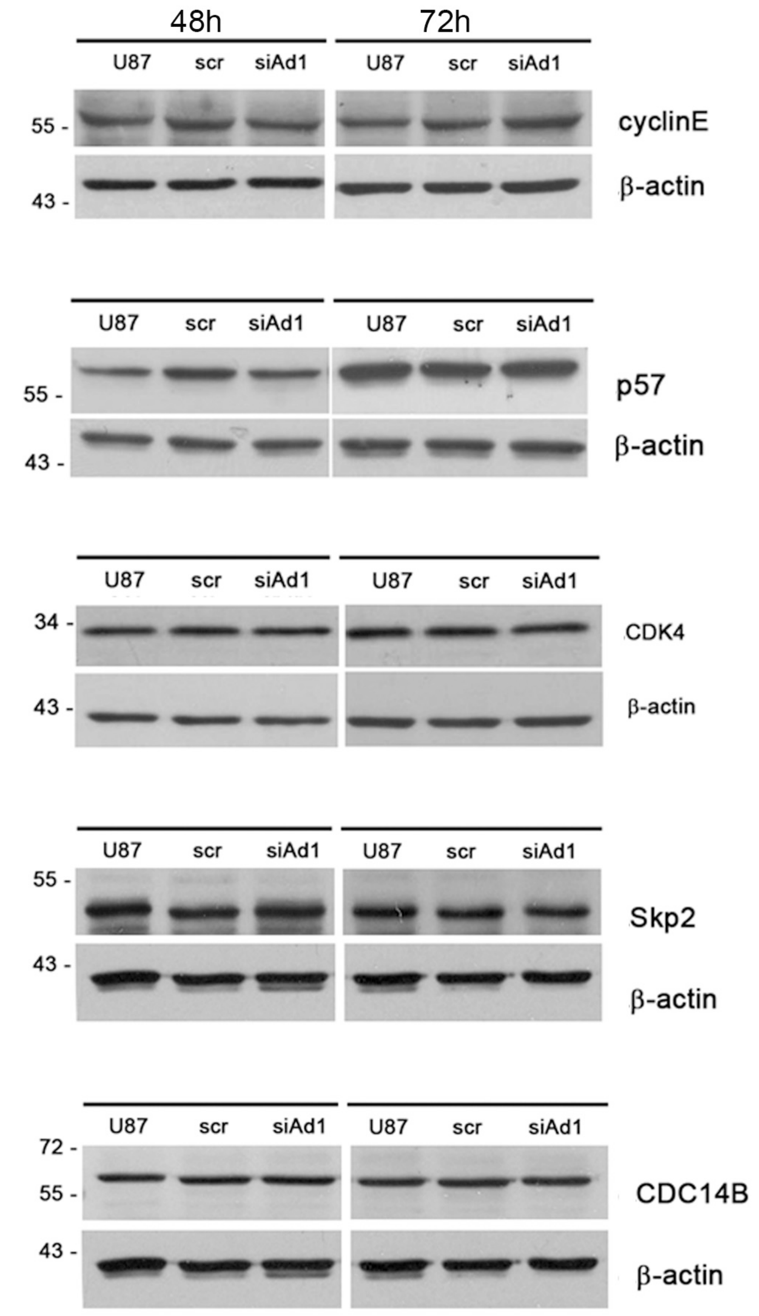

Fig. S7. Protein controlling cell cycle upon ADAR1 silencing. Western blot analysis of different cell cycle regulator proteins (cyclin E, p57, CDK4, Skp2, CDC14B) in siADAR1 (siAd1) U87MG cells at 48 and 72 h post siRNA transfection. β-actin was used as a control.

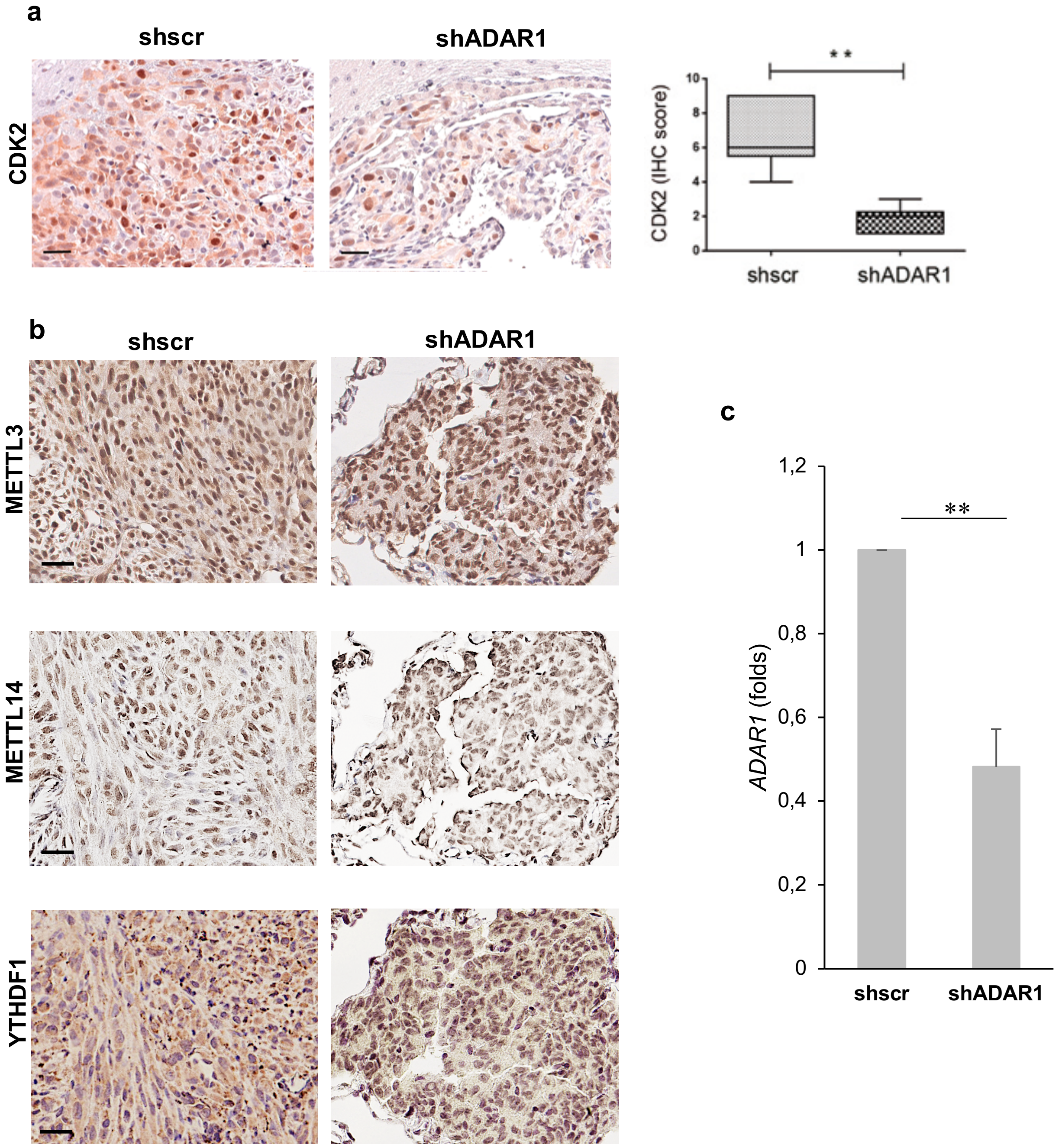

Fig. S8. Orthotopic xenograft of shscr and shADAR1 U87MG cells. Shscr and shADAR1 U87MG cells were injected intracranically; **a)** Left. Representative images showing CDK2 staining in brain tumors obtained after shscr and shADAR1 U87MG cells transplantation. Right. IHC analysis of CDK2 in the same tumors, *p*=0.0022 (Mann-Whitney t-test). **b)** Representative images (IHC) of METTL3, METTL14 and YTHDF1 in orthotopic tumor masses derived from shscr and shADAR1 U87MG cells transplantation. Magnification 40X, Scale bar 25 µm. **c)** qRT-PCR showing ADAR1 mRNA levels in shscr and shADAR1 U87MG-derived orthotopic tumors ***p* value ≤ 0.01.

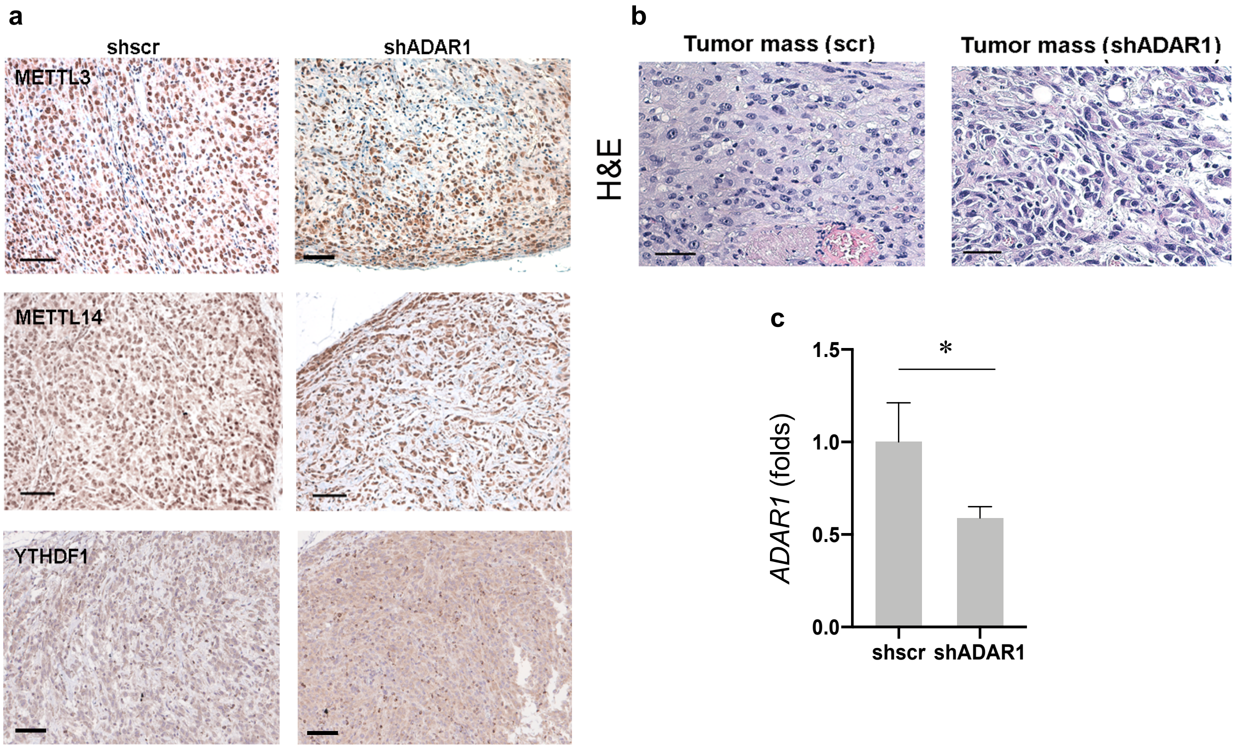

Fig. S9. METTL3, METTL14 and YTHDF1 expression in subcutaneous xenograft of shscr and shADAR1 U87MG cells. a,b) Representative images of IHC showing METTL3, METTL14, YTHDF1 and H&E in subcutaneous tumor masses derived from shscr and shADAR1 U87MG transplantation. Magnification 20X, Scale bar 50 μm. **c)** ADAR1 mRNA levels in shscr and shADAR1 U87MG-derived tumors. *p value ≤ 0.05.
