# Supplementary material for: ADAR1 is a new target of METTL3 and plays a pro-oncogenic role in glioblastoma by an editing-independent mechanism
Source: Genome Biol. 2021 Jan 28;22:51. doi: 10.1186/s13059-021-02271-9 (PMC7842030; doi:10.1186/s13059-021-02271-9)
Supplement: Supplementary file 2 — Additional file 2: Table S1. Patients characteristics. [file 13059_2021_2271_MOESM2_ESM.docx]

| Case | Sex | Age | MGMT methylation status | Tumor location | Surgery | OS (months) | Ki67 (%) | ADAR1 |
| --- | --- | --- | --- | --- | --- | --- | --- | --- |
| GBM1 | M | 59 | Met | Fr | C | 3 | 35 | H |
| GBM2 | M | 72 | Met | Te | P | 8 | 40 | H |
| GBM3 | M | 53 | Unmet | Fr | C | 10 | 30 | H |
| GBM4 | M | 50 | Met | Oc | C | 9 | 25 | H |
| GBM5 | F | 61 | Unmet | Pa | C | 3 | 20 | H |
| GBM6 | M | 54 | Unmet | Oc | C | 6,5 | 15 | H |
| GBM7 | M | 75 | Unmet | Pa | C | 17 | 25 | L |
| GBM8 | F | 70 | Met | Te | P | 9 | 30 | L |
| GBM8 | F | 61 | Unmet | Te | C | 4 | 35 | H |
| GBM9 | M | 63 | Met | Te | C | 19 | 30 | L |
| GBM10 | M | 55 | Unmet | Oc | C | 7 | 35 | L |
| GBM11 | F | 53 | Met | Ot | C | 13 | 15 | L |
| GBM12 | M | 57 | Met | Fr | P | 34 | 15 | L |
| GBM13 | M | 74 | Unmet | Fr | C | 36 | 20 | L |
| GBM14 | M | 51 | Unmet | Pa | C | 13 | 45 | H |
| GBM15 | M | 58 | Unmet | Pa | C | 14 | 40 | H |
| GBM16 | F | 52 | Met | Te | C | 3 | 35 | H |

Abbreviations: GB: glioblastoma; M: male; F: female; Met: methylated; Unmet: unmethylated; Fr: frontal; Te: temporal; Pa: parietal; Oc: occipital; Ot: others; C: complete; P: partial; H: high expression (score 4-9); L: low expression (score 0-3)
