# Supplementary material for: ADAR1 is a new target of METTL3 and plays a pro-oncogenic role in glioblastoma by an editing-independent mechanism
Source: Genome Biol. 2021 Jan 28;22:51. doi: 10.1186/s13059-021-02271-9 (PMC7842030; doi:10.1186/s13059-021-02271-9)

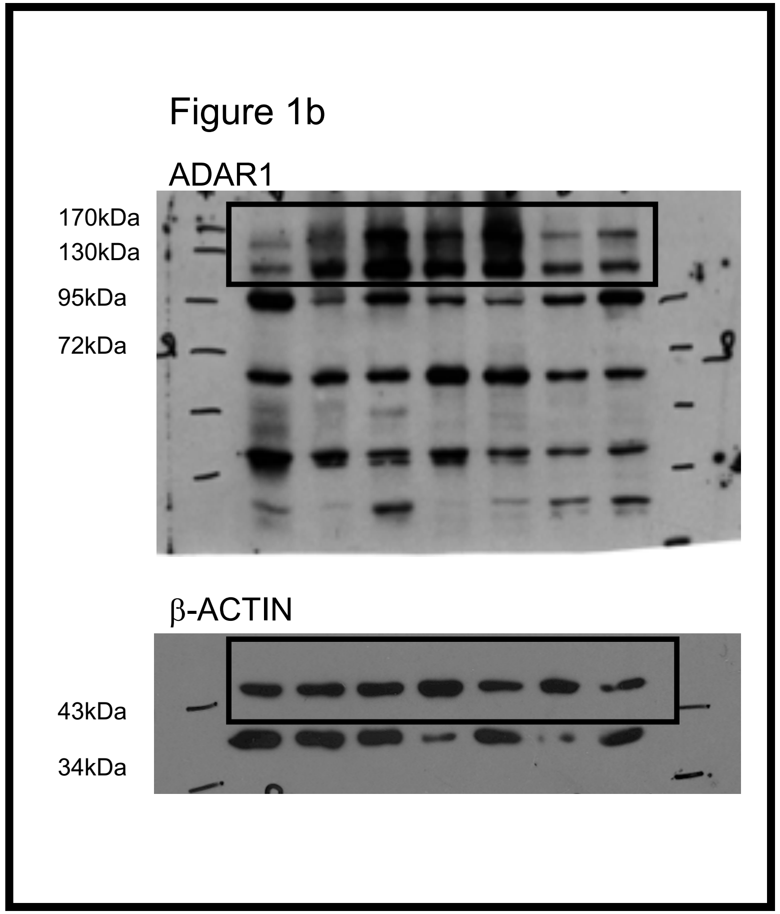
Figure 1b
ADAR1
170kDa
130kDa
95kDa
72kDa
β-ACTIN
43kDa
34kDa

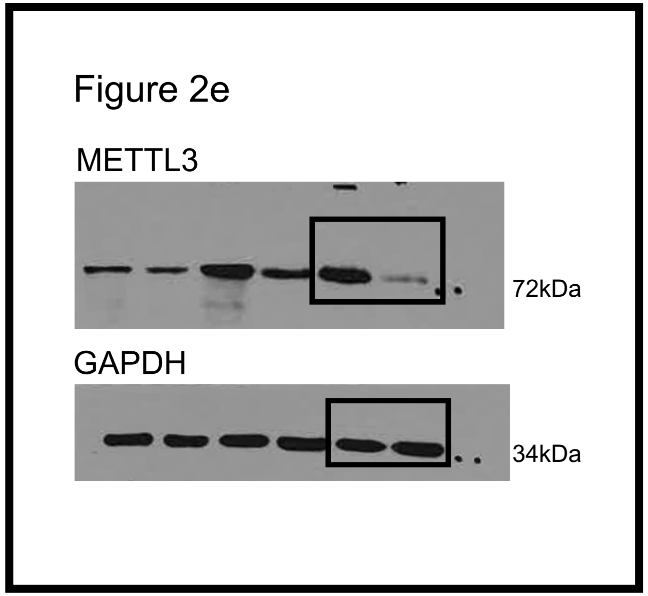
Figure 2e
METTL3
72kDa
GAPDH
34kDa

Figure 3b

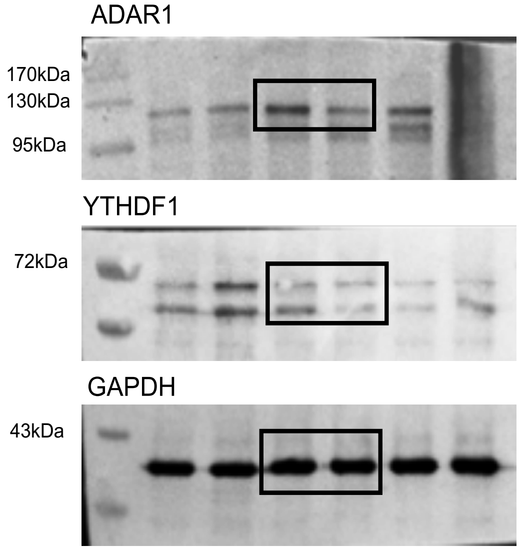


Figure 3c

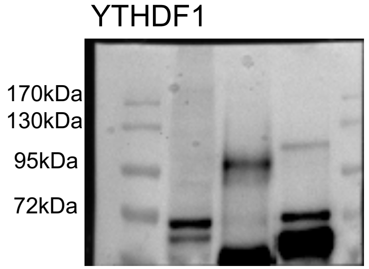


Figure 3d

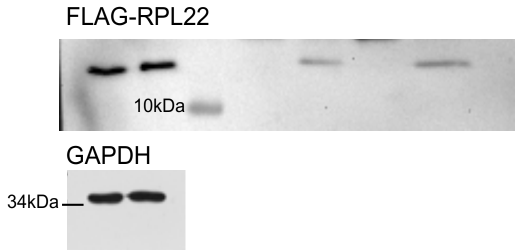


Figure 3f

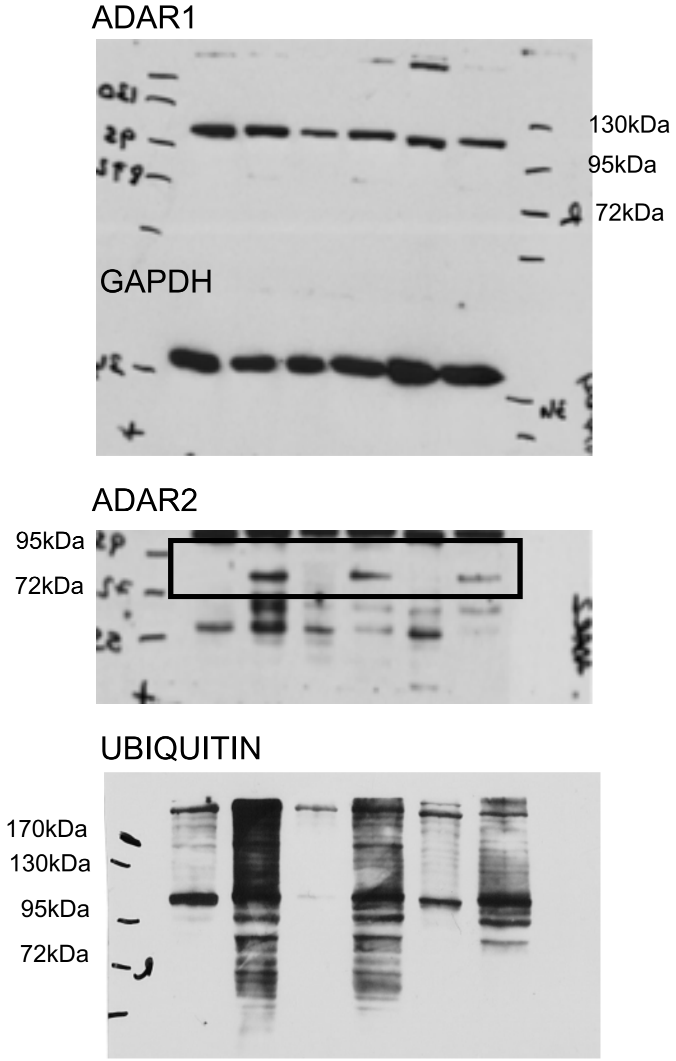


Figure 3f

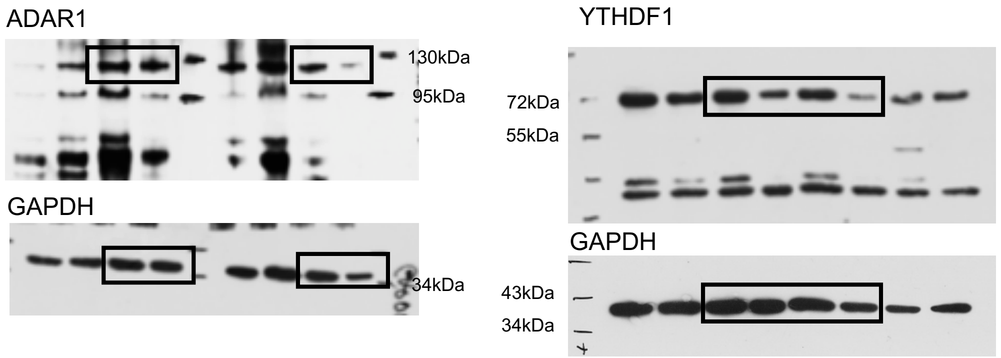

Figure 4a

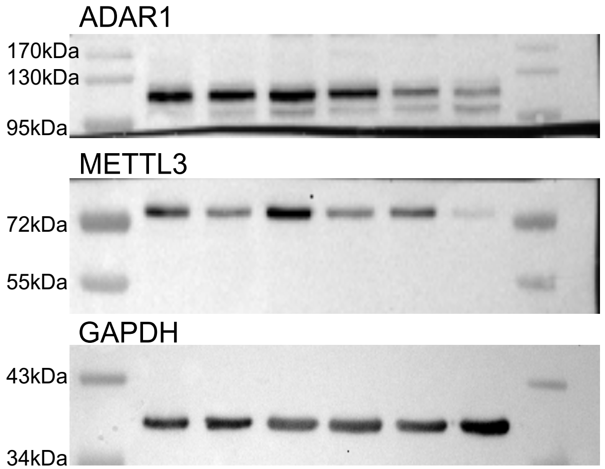


Figure 5b

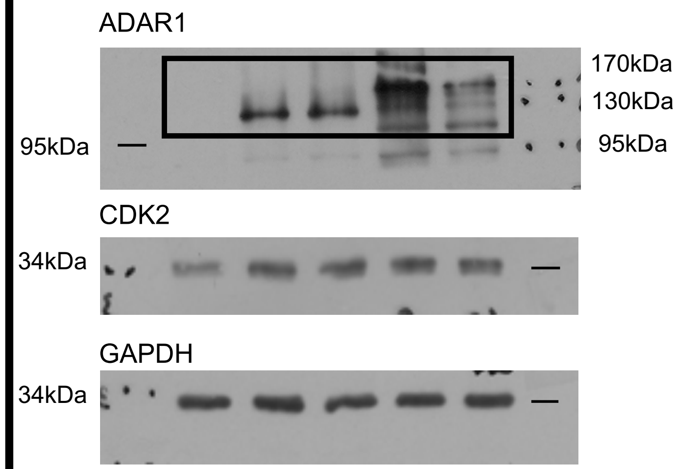


Figure 5d

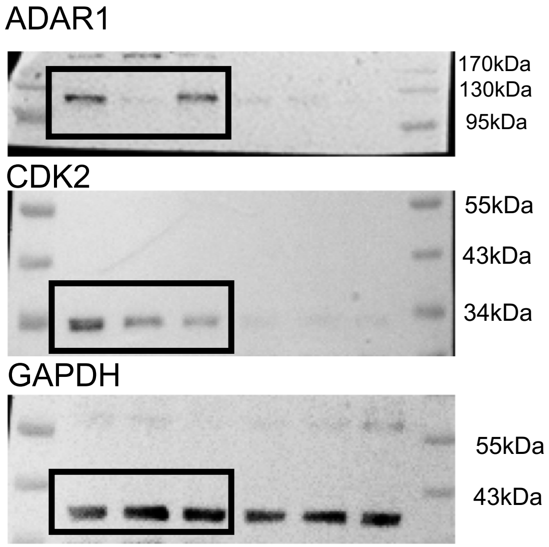


Figure 5e

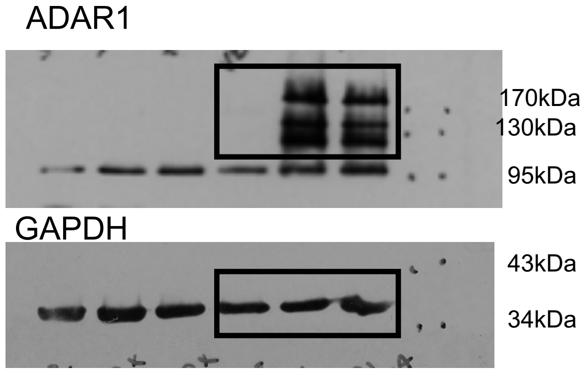

Supplement: Supplementary file 3 — Additional file 3. Uncropped western blotting analysis. [file 13059_2021_2271_MOESM3_ESM.pdf]
