# Supplementary material for: ADAR1 is a new target of METTL3 and plays a pro-oncogenic role in glioblastoma by an editing-independent mechanism
Source: Genome Biol. 2021 Jan 28;22:51. doi: 10.1186/s13059-021-02271-9 (PMC7842030; doi:10.1186/s13059-021-02271-9)
Supplement: Supplementary file 4 — Additional file 4. Review history. [file 13059_2021_2271_MOESM4_ESM.docx]

Review History

**First round of review**

**Reviewer 1**

**Are you able to assess all statistics in the manuscript, including the appropriateness of statistical tests used?** Yes, and I have assessed the statistics in my report.

**Comments to author:**

The manuscript by Tassinari et al deals with the oncogenic role if ADAR1 in glioblastoma. The paper is really interesting and the authors dissect the mechanism using a lot of controls and rescue experiments. Nonetheless, there are few points to be clarified at this stage:

-Figure 1C: please show the protein levels of adar1 in each patients, as has been calculated. Include all the normalizers considered. Protein levels in primary samples can be tricky to quantify. Consider confirming the unaltered mRNA levels.

-Add a table with patients' characteristics.

- fig 2a: did you measure RNA or protein levels? add both

-Fig 2C: what about the localization of MTTL14? Also, METTL14 is never mentioned again in the paper, which is his role in this context?

-Add astrocytes as control for ythdf1 and mettl3 experiments. What happens in normal cells as reference?

-for the in vivo experiments, you should inject parental cells, then treat mice with lentivirus against adar1 alone or in combination with TMZ. In the present form, you just confirmed that cells with shADAR1 are growing less.

-in the in vivo part, add quantification of ADAR1 mRNA, METTL3 and ythdf1 to confirm your model

**Reviewer 2**

**Are you able to assess all statistics in the manuscript, including the appropriateness of statistical tests used?** Yes, and I have assessed the statistics in my report.

**Comments to author:**

Tassinari and colleagues report that upregulation of METTL3 in glioblastoma can methylate ADAR1 mRNA and potentiate its protein level via YTHDF1, thereby promoting tumor growth. The authors argue that the function of ADAR1 is independent of its deaminase activity, but instead depends on binding CDK2 mRNA. Finally, authors found the eligible glioblastoma chemotherapeutic drug-TMZ can achieve a maximum therapeutic effect when combining with Adar1 knockdown. Unfortunately, I found the manuscript to be technically lacking in several critical aspects as I detail in the major concerns below, and thus most of the conclusions made are only based on correlated expression of METTL3, YTHDF1, and ADAR1 in tumors.

Major concerns:

1.The author claim that METTL3 increases ADAR1 protein through YTHDF1. However, the analysis shown in Fig3 cannot support this claim directly. The authors should attenuate METTL3 level and quantify the level of the m6A-marked sites. Experiments involving m6A-seq should be conducted since it's commonly used to evaluated gain or loss of m6A of a particular gene.

2. In continuation with the above, the authors should conduct ribosome-profiling to dissect the mechanism of YTHDF1 in regulating the protein level of ADAR1. It could be either via altering the translational efficacy or decay.

3.The authors claimed that ADAR1 mRNA was bound by YTHDF1 by YTHDF1 immunoprecipitation, but the commercial anti-YTHDF1 antibody does not have a good specificity, thus anti-YTHDF1 immunoprecipitation cannot prove a direct binding of YTHDF1 to ADAR1 mRNA. Besides, the recognition motif of YTHDF1 motif on ADAR1 mRNA should be included.

4.The resolution of IHC is very low that one cannot tell the differential density of signals between groups.

5. It's expected that METTLE and ADAR1 protein can localize in the nucleus, but it doesn't mean these two proteins are interconnected.

6. Since nude mice is a severe immune-deficient mouse, it cannot reflect the tumor progression in a physiological state.

7. Considering the broad impact of METTL3 and YTHDF1 knockdown on tons of mRNA's fate, especially some genes associated with cell cycle, it raises a concern that tumor growth cannot be only attributed to m6A modification in ADAR.

**Authors Response**

**Point-by-point responses to the reviewers’ comments:**

Reviewer #1:

The manuscript by Tassinari et al deals with the oncogenic role if ADAR1 in glioblastoma. The paper is interesting and the authors dissect the mechanism using a lot of controls and rescue experiments.

We would like to thanks the Referee for his/her appreciation regarding our study, we are also pleased that he/she underlined the importance of the study highlighting that we performed experiments rigorously with several controls!

Nonetheless, there are few points to be clarified at this stage:

-Figure 1C: please show the protein levels of adar1 in each patients, as has been calculated. Include all the normalizers considered. Protein levels in primary samples can be tricky to quantify. Consider confirming the unaltered mRNA levels. -Add a table with patients' characteristics.

Following the reviewer’s suggestion, we have now included a new Table (new Additional file 2, Table 1) with the main clinical and pathological features of glioblastoma tumors, also reporting the ADAR1 immunohistochemistry score for each patient as measured by two blinded pathologists that performed the analysis independently. Additionally, we clearly rewrote the materials and methods paragraph relating to the immunoreactivity scoring system. To satisfy the Referee request we also went back to the patients’ bank tissue in order to isolate totRNA from the patients’ samples already tested by IHC, and we performed qRT-PCR analysis for ADAR1. These new data were now included as new Additional file 1, Fig. S1.

-fig 2a: did you measure RNA or protein levels? add both

Thanks to the Referee comment, we have now modified the new Figure 2, reporting the RNA expression level of METTL3 and METTL14 as measured in 163 GBM and 207 brain cortex (http://gepia.cancer-pku.cn/) and performing IHC analysis for both proteins in GBM.

-Fig 2C: what about the localization of MTTL14? Also, METTL14 is never mentioned again in the paper, which is his role in this context?

Following the Referee request, we have now included cell localization of METTL14. In the revised version of the manuscript, we moved all the immunolocalization analysis in Supplementary (Additional file 1, Fig. S2). As METTL14 does not have any catalytic activity, differently from METTL3, we mainly concentrated on METTL3 protein in our study for modulation experiments; however, considering that both are essential components of the m6A methylation core (METLL3/METLL14), we tested whether both these proteins were expressed in glioblastoma (at RNA and protein levels) and their correct cell localization. We included in Result section page 5 the following sentence:

“Therefore, as the core complex of m6A is formed by METTL3 and METTL14, with the first carrying the catalytic subunit and the latter being an essential factor facilitating the RNA binding, we analyzed the presence/level and cell localization of both METTL3 and METTL14 in GBM. We found that both METTL3 and METTL14 are expressed/increased in GBM cells/tissues compared to astrocytes/normal brain cortex with the expected cell localization (Fig. 2a, b, Additional file 1, Fig. S1,2).”

-Add astrocytes as control for ythdf1 and mettl3 experiments. What happens in normal cells as reference?

We already reported that YTHDF1 and METTL3 are expressed in GBM tissues (See Fig. 2 and Fig. 3). Now, following the Referee request, we have performed a set of experiments including western blotting analysis of YTHDF1 and METTL3, METTL14 in normal human primary astrocytes and glioblastoma cell lines (A172, U87 and U118) utilized in our study. The new Figure is now shown as a new Additional file 1, Fig. S1b.

-for the in vivo experiments, you should inject parental cells, then treat mice with lentivirus against adar1 alone or in combination with TMZ. In the present form, you just confirmed that cells with shADAR1 are growing less.

In our study both cell proliferation experiments and xenograft mouse models (orthotopic and not) demonstrated that shADAR1 glioblastoma cells growth less than the control in both in vitro and most importantly in vivo. This is not an obvious result, as many in vitro data failed in the in vivo tests. However, we agree with the Referee, that it is a different story to demonstrate that the ablation of ADAR1 when guided by external stimuli (such as lentivirus, compound, drug, DOXY etc.) still inhibits tumor growth in vivo. To satisfy this specific Referee request and considering that we can perform in vivo experiments under our approved mouse protocol, we decided to inject the not induced glioblastoma cells (either shscr or shADAR1) and then, once the tumor mass started to grow and was measurable, we treated the mice with DOXY (in drinking water) to induce, over time, the expression of shscr and the shADAR1. The induction of ADAR1 silencing, in an in vivo context, worked really well and clearly indicated that ADAR1 is an important target for glioblastoma therapy. The new data were shown as new Figure 7. Considering the strong effect of the induced shADAR1 on tumor growth and that, the shADAR1 tumors, differently to the control mice, were really small and never grew over months (see the new Figure 7 and Figure X for the only benefit of the Referee), the combined regime of shADAR1 with TMZ was unfeasible. However, to satisfy the Referee, as proof of principle experiment and for the only benefit of the Referee, we treated a few mice (randomized) with TMZ (shADAR1 + TMZ) and the results were shown in Figure X. As the shADAR1-tumors are already at their minimum volumes, the effect of the TMZ, even present, was less consistent than the one observed in the control tumors that are highly proliferative and so more sensible to the TMZ action (Figure Xb). In page 11 we have now include the following paragraph:

“Finally, in order to demonstrate that the ablation of ADAR1 when guided by external stimuli (such as lentivirus, compound, drug, DOXY etc.) can inhibit tumor growth in vivo, we injected subcutaneously in the flanks of NOD-SCID mice (n=16) the not induced glioblastoma cells (either shscr or shADAR1) and then, once the tumor mass started to growth, we treated the mice with DOXY (in drinking water) to induce, over time, the expression of shscr and the shADAR1 (Fig. 7a). The induction of ADAR1 silencing in an in vivo context totally blocks tumor growth for over two months (Fig. 7a and data not shown), differently from the control tumor that grows exponentially over time (Fig. 7a). The analysis of METLL3/METLL14 and YTHDF1 expression in these tumors is reported (Additional file 1, Fig. S9) togheter with that of Ki-67, ADAR1 and CDK2 that clearly indicates ADAR1 as an important target for glioblastoma and confirmed the modulation of the ADAR1/CDK2 axis in vivo (Fig. 7b-e). Considering the strong effect of the induced shADAR1 on tumor growth and that, the shADAR1 tumors, differently to the control mice, were really small and never growth over months (Figure 7 and data not shown), the combined regime of shADAR1 with TMZ was unfeasible.”

-in the in vivo part, add quantification of ADAR1 mRNA, METTL3 and ythdf1 to confirm your model.

We have now included in the new version of the manuscript two novel supplementary Figures in Additional file 1, Fig. S8-S9.

**Figure X. Temozolomide treatment of shscr and shADAR1** **U87MG-derived subcutaneous xenografts.** Shscr and shADAR1 U87MG cells were injected subcutaneously into the flanks of nude mice. After about 75 days after transplantation mice were injected i.p. (three times per week for about 3 weeks) with 50 mg/kg of Temozolomide. Quantitative analysis of tumors is shown as **a** tumor size (mm3) and **b** percentage of tumor volume.

**Methods:**

For the Temozolomide (TMZ) treatment, 4 mice in each group (shscr and shADAR1) were randomized and the control group was treated with TMZ solvent (DMSO) while the other with 50 mg/kg of TMZ, three times/week for about 3 weeks. Tumor size was assessed by caliper measurement and tumor volume was calculated as described above.

Reviewer #2:

Tassinari and colleagues report that upregulation of METTL3 in glioblastoma can methylate ADAR1 mRNA and potentiate its protein level via YTHDF1, thereby promoting tumor growth. The authors argue that the function of ADAR1 is independent of its deaminase activity, but instead depends on binding CDK2 mRNA. Finally, authors found the eligible glioblastoma chemotherapeutic drug-TMZ can achieve a maximum therapeutic effect when combining with Adar1 knockdown. Unfortunately, I found the manuscript to be technically lacking in several critical aspects as I detail in the major concerns below, and thus most of the conclusions made are only based on correlated expression of METTL3, YTHDF1, and ADAR1 in tumors.

We are sorry that we were unable to stress the importance of the study that is far from being a descriptive study as it includes multiple experimental approaches and controls. Considering the Referee comments, we have tried, in the revised version of the study, to underline the importance of all the experiments we did that, together with the novel set of data requested by the Referees, disclosed a novel biological pathway interconnecting m6A and ADAR1 that was never shown before, furthermore we have also shown that ADAR1, independently of its deaminase activity, acts as an oncogene and it is an important target for glioblastoma therapy!

Major concerns:

1.The author claim that METTL3 increases ADAR1 protein through YTHDF1. However, the analysis shown in Fig3 cannot support this claim directly. The authors should attenuate METTL3 level and quantify the level of the m6A-marked sites. Experiments involving m6A-seq should be conducted since it's commonly used to evaluated gain or loss of m6A of a particular gene.

Thanks to the Referee, we have performed a new set of experiments in which we modulated METTL3 expression level and quantified the level of ADAR1 m6A-marked sites. Our data showed that m6A-ADAR1 significantly decreased upon METTL3 downregulation. The new data were now shown as a new Figure 2e-f. Figure 2 legend, Results and Discussion were modified accordingly and the new text is shown in red in the revised manuscript. Additionally, we downloaded the m6A-seq from shMETTL3/METTL14 and control glioma stem cells (Cui et al, 2017), and we re-analyzed the data concentrating on ADAR1 transcript. We found a reduction of ADAR1 methylated regions (including the ones studied in the present study, Chr1:154557253-7418) in the shMETTL3/14 samples (data shown in Fig. Y for the only benefit of the Referee). The above findings were included in Result section (page 6) text in red: “Then, we modulated METTL3 expression (shMETTL3) in glioblastoma cells and we quantified the m6A-marked sites of ADAR1 by qRT-PCR. Our data demonstrated that the m6A-RNA fragments of ADAR1 (PCR1-2) were significantly decreased upon METTL3 silencing (Fig. 2e-f). Finally, we downloaded the m6A-seq from shMETTL3/METTL14 and control glioma stem cells [13], and we analyzed the data concentrating on ADAR1 transcript, founding a reduction of ADAR1 methylation levels (including the ones studied in the present study, Chr1:154557253-7418) in both the shMETTL3 and shMETTL14 samples (data not shown).”

GSC m6A-seq data on ADAR1 (from Cui et al, 2017)

Silencing METTL3

| **chr** | **chromStart** | **chromEnd** | **name** | **score** | **strand** | **blockCount** | **blockSizes** | **blockStarts** | **lg.p** | **lg.fdr** | **fold_enrchment** | **diff.lg.fdr** | **diff.lg.p** | **diff.log2.fc** |
| --- | --- | --- | --- | --- | --- | --- | --- | --- | --- | --- | --- | --- | --- | --- |
| chr1 | 154557201 | 154557412 | ADAR | 1.00E-26 | - | 1 | 211, | 0 | -26 | -24.5 | 2.59 | -0.208 | -1.11 | -0.41 |
| chr1 | 154570395 | 154571031 | ADAR | 5.00E-35 | - | 2 | 57,154, | 0,482 | -34.3 | -32.7 | 2.42 | -0.208 | -0.685 | -0.191 |
| chr1 | 154573695 | 154574176 | ADAR | 2.00E-55 | - | 1 | 481, | 0 | -54.7 | -52.9 | 3.78 | -0.208 | -0.633 | -0.137 |
| chr1 | 154574475 | 154574716 | ADAR | 3.20E-11 | - | 1 | 241, | 0 | -10.5 | -9.26 | 1.69 | -0.208 | -1.28 | -0.381 |
|  |  |  |  |  |  |  |  |  |  |  |  |  |  |  |

Silencing METTL14

| **chr** | **chromStart** | **chromEnd** | **name** | **score** | **strand** | **blockCount** | **blockSizes** | **blockStarts** | **lg.p** | **lg.fdr** | **fold_enrchment** | **diff.lg.fdr** | **diff.lg.p** | **diff.log2.fc** |
| --- | --- | --- | --- | --- | --- | --- | --- | --- | --- | --- | --- | --- | --- | --- |
| chr1 | 154557172 | 154557412 | ADAR | 4.00E-49 | - | 1 | 240, | 0 | -48.4 | -46.8 | 2.58 | -0.223 | -1.03 | -0.333 |
| chr1 | 154570365 | 154571031 | ADAR | 7.90E-70 | - | 2 | 87,154, | 0,512 | -69.1 | -67.4 | 2.59 | -0.203 | -0.303 | 0.0128 |
| chr1 | 154573695 | 154574176 | ADAR | ######## | - | 1 | 481, | 0 | -100 | -98.5 | 3.81 | -0.203 | -0.798 | -0.166 |
| chr1 | 154574415 | 154574776 | ADAR | 1.30E-36 | - | 1 | 361, | 0 | -35.9 | -34.5 | 2.13 | -0.203 | -0.449 | 0.073 |

shMETTL3

shMETTL14

2. In continuation with the above, the authors should conduct ribosome-profiling to dissect the mechanism of YTHDF1 in regulating the protein level of ADAR1. It could be either via altering the translational efficacy or decay.

To satisfy to the Referee request, we set up new experiments spanning from translation to RNA/protein decay. We were able to demonstrate that m6A machinery modulates ADAR1 protein level through translation without affecting RNA or protein decay. The new data are now part of a novel Figure 3. Figure legend, Results and Discussion were modified accordingly and the new text is shown in red in the revised manuscript. Specifically, for the ribosome-profiling we took advantage of a highly precise experimental approach previously used in top journal publication (Rapino et al, Nature 2018) that utilizes the RPL22–Flag construct (Ribosomal Protein L22). We performed ribosome immunoprecipitation experiments using U87MG human cells overexpressing the FLAG-tagged RPL22 (ribosomal protein L22) in order to quantify the ribosome occupancy of ADAR1 mRNAs in shYTHDF1 and control. Notably, we found that ribosomes accumulate ADAR1 mRNAs in control cells while reduce ADAR1 mRNAs upon YTHDF1 depletion (new Fig. 3d). We have now included the following sentence in page 7:

“In order to quantify ribosome occupancy of ADAR1 mRNAs, mediated by its m6A modification, we performed ribosome immunoprecipitation experiments using U87MG human cells that expressed Flag-tagged RPL22 (ribosomal protein L22) upon YTHDF1 modulation (Rapino et al, Nature 2018). Notably, we found that ribosomes accumulate ADAR1 mRNAs in control cells, while a reduced ADAR1 mRNAs ribosome occupancy was observed in YTHDF1 silenced cells (Fig. 3d and Additional file 1, Fig S3).”

The Referee asked whether “It could be either via altering the translational efficacy or decay.”

To investigate whether YTHDF1 can mediate ADAR1 RNA decay, we performed a new set of experiments in which we silenced YTHDF1 with or without actinomycin D and we measured the level of ADAR1 (qRT-PCR). The new data demonstrated that YTHDF1 silencing did not affect ADAR1 RNA decay. Furthermore, we also investigated whether protein decay can play a role in this scenario. To investigate this possibility, we silenced YTHDF1 with or without MG132 (proteasome inhibitor) and we measured the protein level of ADAR1, demonstrating, once more, that METLL3/ YTHDF1 axis has the ability to modulate ADAR1 protein level independently of protein decay. These new set of data are now shown as new Fig. 3e-f. We have now included a new paragraph, page 7: “To determine whether the expression level of ADAR1 is not correlated with changes in mRNA stability upon YTHDF1 modulation, we measured the levels of ADAR1 mRNA after transcription inhibition with actinomycin D. We show that ADAR1 mRNA stability was unaffected upon YTHDF1depletion (Fig. 3e). Therefore, to investigate whether protein decay can play a role in this scenario, we silenced YTHDF1 and treated the cells with or without MG132 (proteasome inhibitor), and then we measured ADAR1protein level. ADAR2 (Rossetti et al, 2017) and anti-ubiquitin antibody were used as controls. We show that YTHDF1 can modulate ADAR1 protein independently of protein degradation mechanism (Fig. 3f).”

3. The authors claimed that ADAR1 mRNA was bound by YTHDF1 by YTHDF1 immunoprecipitation, but the commercial anti-YTHDF1 antibody does not have a good specificity, thus anti-YTHDF1 immunoprecipitation cannot prove a direct binding of YTHDF1 to ADAR1 mRNA.

Besides, the recognition motif of YTHDF1 motif on ADAR1 mRNA should be included.

In our hands the Ab against YTHDF1 (Abcam, Ab99080) works well, indeed the antibody specificity was also shown by the internal control (HPRT) that was not altered (differently from ADAR1 mRNA) in the RIP experiments. However, to satisfy the Referee we have used a second antibody against YTHDF1 (and Ab220162) and we have repeated the experiments including an internal control. Again, we found that the ADAR1 mRNA was specifically immunoprecipitated while the control RNAs did not. The new data are now part of a new Fig.3. Figure legend, Results and Discussion were modified accordingly and the new text is shown in red in the revised manuscript.

Regarding the “Besides, the recognition motif of YTHDF1 motif on ADAR1 mRNA should be included.”

As far as we know, there is not any recognition motif for YTHDF1. YTHDF1 recognizes m6A on RNA transcripts, instead of motifs that co-exist in the proximity of m6A sites (Wang et al., 2005).

4.The resolution of IHC is very low that one cannot tell the differential density of signals between groups.

Thanks to the Referee, we realized that some IHCs lost their original magnification during the Figure construction. We have corrected it.

5. It's expected that METTLE and ADAR1 protein can localize in the nucleus, but it doesn't mean these two proteins are interconnected. Correct! The IF were reported to give a general summary of METTL3 and ADAR1 protein expression/localization in glioblastoma cells. We have now moved the IF panels to Supplementary (Additional file 2, Fig. S2). We have better clarify this point in Result page 5: “Therefore, as the core complex of m6A is formed between METTL3 and METTL14, with the first carrying the catalytic subunit and the latter being an essential factor facilitating the RNA binding, we analyzed the presence/level and cell localization of both METTL3 and METTL14 in GBM. We found that both METTL3 and METTL14 are expressed/increased in GBM cells/tissues compared to astrocytes/normal brain cortex with the expected cell localization (Fig. 2a, b, Additional file 2, Fig. S1,2).”

6. Since nude mice are a severe immune-deficient mouse, it cannot reflect the tumour progression in a physiological state. In our study, we used a xenograft model so that tumour cells from a species (human) will be injected/transplanted in a different recipient species (mice). In order to pre¬vent rejection, the cells of the host’s immune system have necessarily to be impaired. Tumor xenografts are model for tumor growth utilized worldwide for years until the present time, and results of these models were translated in over 4000 manuscripts in pubmed (see as example Shafi Kuchay et al, Nature 2017; Dohoon Kim et al, Nature 2015; Yugang Wang at el, Nature 2017; Chunming Cheng et al, Cancer Cell 2015; Li W, et al, J Exp Clin Cancer Res. 2018; Jin F,.et al, Pharm Biol. 2018; Li J,et al, J Exp Clin Cancer Res. 2018; Hwang JY, et al, .Cancer Lett. 2019 ; Xu D, et al, .Cell Death Dis. 2018 ; Delwar ZM,et al, Cancer Res. 2018). Thanks to these models (xenograft) a huge amount of data were collected, so far, clarifying mechanisms for tumor growth and gave essential information for preclinical and clinical studies. Both retrospective and prospective studies revealed that human tumor xenografts can be remarkably predictive of the tumor growth and, for this reason were also utilized for cytotoxic chemotherapeutic drugs that have activity in humans. Considering that in our study we want to demonstrate, for the first time, that targeting ADAR1 in vivo can inhibit glioblastoma growth, we believe that the model we utilized is more than appropriate at this stage. Of note, in the recent years it has been developed a novel NOD/SCID mice model that can be “humanized” by injecting peripheral blood or bone marrow cells into the mice, allowing for an almost complete reconstitution of the immune response to the tumor. However, the above model mainly aimed at studying the interconnection of tumor and immunological cells, crosstalk that is extremely limited in GBM differently to many other type of cancer.

7. Considering the broad impact of METTL3 and YTHDF1 knockdown on tons of mRNA's fate, especially some genes associated with cell cycle, it raises a concern that tumor growth cannot be only attributed to m6A modification in ADAR.

We never stated in our manuscript that the cell cycle phenotype due to METTL3/YTHDF1 knockdown is exclusively mediated by ADAR1 neither that this is true in all the cell types and/or cancer types!

Specifically, in several parts of our manuscript, we stated that ADAR1 is one of the downstream players of METTL3/YTHDF1, and our data, at least in glioblastoma, pointed to ADAR1 as one of the main players.

Indeed, in the Conclusion section, Page 15, we clearly stated: “We demonstrated that ADAR1 is one of the main targets of N6-Methyladenosine METTL3 controlling cell proliferation in glioblastoma”

Of note, ADARs affect over 15.000.000 sites in humans (Mansi et al, 2020). Specifically, ADAR1 modulates tons of mRNAs, microRNAs, and lncRNAs (Jae Hoon Bahn et al 2015, Oz Solomon et al, 2017, Samuel 2019, Wu et al, 2019, Silvestris et al, 2020, etc) with some of these involved in the cell cycle progression (Qingfei Jiang et al, 2019), as a consequence, the modulation of ADAR1 protein has a huge impact on the human transcriptome.

**Second round of review**

**Reviewer 1**

The authors modified the manuscript according to the reviewer comments.

This manuscript is of great interest for the readers of the journal.

**Reviewer 2**

1: My major concern is whether the observation of ADAR protein accumulation in GBM samples could be explained by RNA m6A-YTHDF1 axis, which facilitates mRNA translation. The authors’ data show that GBM have higher expression of METTL3 and YTHDF1 in both RNA and protein levels, ADAR have m6A sites and could be bound by YTHDF1. But they didn’t discuss other well-known m6A readers at all.

I suggested they at least showed similar figures as Fig2a/3a to show the RNA levels of YTHDF2 and YTHDF3. Do these m6A reader also increased in GBM? If the answer is yes, the authors need to perform knock-out/ knock-down assay to dissect the downstream regulatory mechanism of m6A sites in ADAR. In addition, according to GEPIA database, did the RNA levels of ADAR also increased GBM samples?

2: To answer my previous question 3, the authors claimed that the Antibody works well. To support this, they need to show the YTHDF1 antibodies (both Ab99080 and Ab220162) won’t bind to YTHDF2 and YTHDF3.

**Authors Response**

**Point-by-point responses to the reviewers’ comments:**

Point-by-point

Reviewer #2: 1: My major concern is whether the observation of ADAR protein accumulation in GBM samples could be explained by RNA m6A-YTHDF1 axis, which facilitates mRNA translation. The authors’ data show that GBM have higher expression of METTL3 and YTHDF1 in both RNA and protein levels, ADAR have m6A sites and could be bound by YTHDF1. But they didn’t discuss other well-known m6A readers at all. I suggested they at least showed similar figures as Fig2a/3a to show the RNA levels of YTHDF2 and YTHDF3. Do these m6A reader also increased in GBM? If the answer is yes, the authors need to perform knock-out/ knock-down assay to dissect the downstream regulatory mechanism of m6A sites in ADAR. In addition, according to GEPIA database, did the RNA levels of ADAR also increased GBM samples?

*In our manuscript we have clearly demonstrated that METTL3 is able to increase ADAR1 protein. Additionally, we also investigate the involvement of the reader YTHDF1 in this pathway, as this specific protein is significantly upregulated in glioblastoma compared to controls (Fig. 3a and Additional file 1, Fig. S1b), and was already reported, among the other YTH members, as the most highly upregulated reader protein in GBM samples (582 samples) compared with normal brain samples (720 samples) (Yarmishyn et al 2020).*

*Exploring all possible molecular mechanisms implicated in ADAR1 modulation is beyond the scope of the present study. Further studies will be necessary to investigate additional players involved in the m6A ADAR1 transcript binding and modulation.*

*In Result section page 6-7 we have added the following sentence:*

*“Interestingly, we observed that YTHDF1 was increased in GBM cells and tissues compared to controls (Fig. 3a and Additional file 1, Fig. S1b). Additionally, a recent study reported that, among the five YTH family members, the YTHDF1 was found to be the most highly upregulated in GBM samples (582 samples) compared with normal brain samples (720 samples) (Yarmishyn et al 2020).”*

*Regarding ADAR1 RNA level in GBM and normal brain we have already published this data in our previous study (Silvestris et al, 2019) as also clearly indicated in our manuscript (page 5, line 6). Specifically, we analysed RNA-Seq from 145 primary GBMs (TCGA dataset) compared to 132 RNA-Seq (GTEx project) from the normal brain cortex and we found that no significant difference of ADAR1 RNA expression was observed. Similar results were found when we interrogated GEPIA database (data not shown).*

2: To answer my previous question 3, the authors claimed that the Antibody works well. To support this, they need to show the YTHDF1 antibodies (both Ab99080 and Ab220162) won’t bind to YTHDF2 and YTHDF3.

*Regarding the Referee 2 concerns of the YTHDF1 antibodies specificity, we would like to stress the two different antibodies were guaranteed by the Company (Abcam) for their specific, as the Company tested them using extensive validation methods, including knockout validation (as indicated in the Abcam data sheets). Additionally, these specific antibodies has been widely used in several papers published in*

*prestigious journals in which the authors were able to clearly distinguish among the different YTHDF1/2/3-proteins (as example: Ding H et al. GNAS promotes inflammation-related hepatocellular carcinoma progression by promoting STAT3 activation. Cell Mol Biol Lett 25:8 (2020); Human. Jin D et al. m6A demethylase ALKBH5 inhibits tumor growth and metastasis by reducing YTHDFs-mediated YAP expression and inhibiting miR-107/LATS2-mediated YAP activity in NSCLC. Mol Cancer 19:40 (2020); Human; Zhao W et al. METTL3 Facilitates Oral Squamous Cell Carcinoma Tumorigenesis by Enhancing c-Myc Stability via YTHDF1-Mediated m6A Modification. Mol Ther Nucleic Acids 20:1-12 (2020); Human; Huang B et al. Cyclophosphamide Regulates N6-Methyladenosine and m6A RNA Enzyme Levels in Human Granulosa Cells and in Ovaries of a Premature Ovarian Aging Mouse Model. Front Endocrinol (Lausanne) 10:415 (2019)).*

*Additionally, in our study, we have already shown that the specific silencing of the YTHDF1 eliminated the WB band detected by both these antibodies (Figure 3 and data not shown), indeed considering that all the YTHDF1, 2 and 3 run at the same MW (70kDa) it would be really incredible if two different antibodies and a siRNA were all found to be non-specific.*

*Despite the above, we performed the WB analysis using U87 cells silenced for YTHDF1 and tested the Ab Ab220162 that was indeed used for all the experiments show in Fig 3c-f, togheter with Abs YTHDF2 and YTHDF3. We did not retested the Ab99080 because the siYTHDF1 with this antibody has been already shown in Fig. 3b. The WB demonstrated that also this Ab is specific for YTHDF1.*

*wb showing the specificity of ythdf1 antibody in U87MG 48 hours post YTHDF1 silencing.*
